# Easy Synthesis of Complex Biomolecular Assemblies: Wheat Germ Cell-Free Protein Expression in Structural Biology

**DOI:** 10.3389/fmolb.2021.639587

**Published:** 2021-03-25

**Authors:** Marie-Laure Fogeron, Lauriane Lecoq, Laura Cole, Matthias Harbers, Anja Böckmann

**Affiliations:** ^1^ Molecular Microbiology and Structural Biochemistry, Labex Ecofect, UMR 5086 CNRS/Université de Lyon, Lyon, France; ^2^ CellFree Sciences, Yokohama, Japan; ^3^ RIKEN Center for Integrative Medical Sciences (IMS), Yokohama, Japan

**Keywords:** cell-free protein expression, wheat germ, structural biology, NMR, labeling

## Abstract

Cell-free protein synthesis (CFPS) systems are gaining more importance as universal tools for basic research, applied sciences, and product development with new technologies emerging for their application. Huge progress was made in the field of synthetic biology using CFPS to develop new proteins for technical applications and therapy. Out of the available CFPS systems, wheat germ cell-free protein synthesis (WG-CFPS) merges the highest yields with the use of a eukaryotic ribosome, making it an excellent approach for the synthesis of complex eukaryotic proteins including, for example, protein complexes and membrane proteins. Separating the translation reaction from other cellular processes, CFPS offers a flexible means to adapt translation reactions to protein needs. There is a large demand for such potent, easy-to-use, rapid protein expression systems, which are optimally serving protein requirements to drive biochemical and structural biology research. We summarize here a general workflow for a wheat germ system providing examples from the literature, as well as applications used for our own studies in structural biology. With this review, we want to highlight the tremendous potential of the rapidly evolving and highly versatile CFPS systems, making them more widely used as common tools to recombinantly prepare particularly challenging recombinant eukaryotic proteins.

## Introduction

Efficient, easy-to-use, and rapid protein expression methods for protein analysis are in great demand for structural determination, biochemical research, and applications in synthetic biology, such as the design of new biological circuits or the development of new proteins for technical applications and therapies (Gregorio et al., 2019; Silverman et al., 2020). The rapid response to the recent COVID-19 pandemic shows how the scientific community is applying the latest technologies to study viral proteins and to make them available for structural analysis (Zhu et al., 2020b) and drug testing (Dai et al., 2020; Jin et al., 2020), the development of antibodies, or creation of new serological tests to monitor infection rates. In this context, cell-free protein synthesis (CFPS) was used to make versions of the SARS-CoV-2 N-protein using wheat germ cell-free protein synthesis (WG-CFPS) for use in serological testing (Matsuba et al., 2020; Yamaoka et al., 2020) and antibody development leading to tests for COVID-19 now available on the market to serve patients. Also, a variety of accessory and structural proteins have been synthesized and purified in milligram amounts using this approach (Altincekic et al., 2021)^1^. CFPS platforms in general are versatile tools to address such needs (Rosenblum and Cooperman, 2014), building on previous work on pathogen-related research (Matsunaga et al., 2014; Yamaoka et al., 2016). These protein expression platforms can be customized to work on individual proteins or have been scaled for high-throughput protein expression for analysis and production on large scales. Hence, CFPS (recently also called TXTL for “transcription-translation”) is getting more attention these days with the development of new methods that try to make the best use of the unique features of an *in vitro* method rather than relying on established systems depending on a host cell (Zemella et al., 2015) for synthesis of recombinant proteins. The high potential of new CFPS systems was demonstrated by an *E. coli* system that is used for protein expression on an industrial scale (Zawada et al., 2011; Salehi et al., 2016; Hershewe et al., 2020). It was suggested that such systems could be used more in the future for the production of dedicated pharma proteins, for example, incorporating noncanonical amino acids (Hong et al., 2014; Quast et al., 2015; Wu et al., 2020), preparation of dedicated proteins that were produced under more defined conditions than possible in cell-based systems (Oza et al., 2015), or allowing for the “on-demand” production of protein therapeutics in the clinic (Mohr et al., 2016; Sullivan et al., 2016; Timm et al., 2016). The diverse features of CFPS systems promoted also their recent use in teaching (Stark et al., 2019), protein engineering (Kido et al., 2020), and synthetic biology (Tinafar et al., 2019), which holds great promises for studies on genetic networks or rapid prototyping (Karim et al., 2020) in metabolic engineering (Perez et al., 2016) as well as future drug development (Dondapati et al., 2020). Moreover, the *in vitro* reaction format of CFPS systems allows for full automation, miniaturization (Ayoubi-Joshaghani et al., 2020), and working with large sample numbers (Zhu et al., 2015). This advantage has been utilized in large-scale screening experiments (Khnouf et al., 2010; Kim et al., 2015), searches for malaria vaccine candidates (Kanoi et al., 2017; Morita et al., 2017; Kanoi et al., 2020), identifying interactions between E3 ligases and their substrates (Takahashi et al., 2016), building a protein array holding human Deubiquitinating Enzymes (DUBs) (Takahashi et al., 2020), or the development of protein array platforms (Romanov et al., 2014; Zarate and Galbraith, 2014; Morishita et al., 2019). Other promising developments make use of the stability of the reagents, where the extracts and buffers can be lyophilized for long-term storage at room temperature (Smith et al., 2014). This enabled the development of a paper-based diagnostic assay for the detection of Ebola (Pardee et al., 2014), a concept that could be extended to the development of more sensitive rapid tests for other infectious diseases suitable for use in developing countries or testing water quality (Jung et al., 2020) with a simple assay (Grawe et al., 2019; Thavarajah et al., 2020). It is a promising approach to combine DNA detection with the expression of a marker protein, which will enable new concepts for biosensor developments (Duyen et al., 2016; Ogawa et al., 2016; Zhang et al., 2020). For such applications, the translation system could also be miniaturized or used in a fluidic array device (Jackson et al., 2015) for automation and easy use.

Whether used in high-throughput or on individual proteins, CFPS systems can be optimized in ways not possible for cell-based systems. The open nature of an *in vitro* reaction allows for changes to the reaction environment to mimic better individual protein needs. This was demonstrated in many studies for the most commonly used commercial or self-made CFPS systems from *E. coli* using customized extract preparations on a large variety of proteins for different applications (Gregorio et al., 2019; Cole et al., 2020). Another well-established system on which we will focus here is based on wheat germ extracts (Roberts and Paterson, 1973; Madin et al., 2000). Eukaryotic ribosomes from plants are better adapted for protein folding during synthesis than prokaryotic ribosomes from *E. coli* extracts, notably when eukaryotic proteins are targeted. Besides those established CFPS systems (Rosenblum and Cooperman, 2014; Zemella et al., 2015; Dondapati et al., 2020), new systems were developed for rapid protein expression that better match the features of cell-based systems, for instance, using extracts from HeLa (Mikami et al., 2008) or Chinese Hamster Ovary (CHO) cells (Brodel et al., 2015; Thoring et al., 2016). Other advancing systems are based on extracts from *Saccharomyces cerevisiae* (Gan and Jewett, 2014), *Pichia pastoris* (Spice et al., 2020), tobacco BY-2 cells (Buntru et al., 2015), rice (Suzuki et al., 2020), or modified *E. coli* strains (Seki et al., 2009; Cole et al., 2020) to name a few. Our growing understanding of translation reactions and a deeper understanding of the cell extracts led to new protocols for extracts having improved activity (Borkowski et al., 2020; Contreras-Llano et al., 2020) or been engineered for specialized applications such as working better with noncanonical amino acids (Martin et al., 2018). All those modern CFPS systems have often been optimized for high protein yields and better cost performance, thus by far exceeding the abilities of the classical rabbit reticulocyte lysate system that is still widely used in protein labeling reactions and biochemical studies. Among the eukaryotic systems, high-performance wheat germ extracts have shown the highest protein expression activity (Perez et al., 2016), leading to the wide use of this system in research and applied sciences. Since the germ is in a dormant stage, it is an extraordinarily rich source for the protein factors and the ribosomes needed for rapidly performing protein synthesis from stored mRNAs during early germination (Sano et al., 2020).

In the context of structural biology, protocols developed for the preparation of highly active wheat germ extracts lead to a universal protein expression system (Sawasaki et al., 2002b) that is used in a variety of structural approaches, such as preparing stable-isotope-labeled samples for protein NMR (Lacabanne et al., 2019), making reference standards for mass spectrometry in proteomics (Singh et al., 2009; Takanori et al., 2017), or preparing samples for cryogenic electron microscopy (cryo-EM) (Novikova et al., 2018). Notably, sample amounts needed in structural biology have significantly diminished in the last years with the development of crystallization robots for X-ray studies, high-performance detectors in cryo-EM, and higher magnetic fields in NMR (Dobson, 2019). Particularly, in solid-state NMR, faster magic-angle spinning (MAS) recently reduced sample needs by a spectacular factor of 100 through proton detection under MAS frequencies exceeding 100 kHz (Agarwal et al., 2014; Bockmann et al., 2015; Lecoq et al., 2018; Lecoq et al., 2019; Wang et al., 2019), a milestone that enables investigation of submilligram amounts of sample. As solid-state NMR can typically target large protein assemblies such as viral capsids (Zhang et al., 2016; Wang et al., 2017; Quinn et al., 2018), envelopes (David et al., 2018), microtubules (Guo et al., 2019), or membrane proteins (Jirasko et al., 2020) and their assemblies (Ong et al., 2013; Kaur et al., 2015; Kaur et al., 2016; Kaur et al., 2018; Kaur et al., 2019), an *in vitro* protein synthesis system using a high-yielding eukaryotic ribosome is a central asset for such studies. This approach can generally be used to also produce proteins of pathogens that hijack the eukaryotic host cell machinery during infections making it a powerful tool for pathogen research.

**FIGURE 1:**
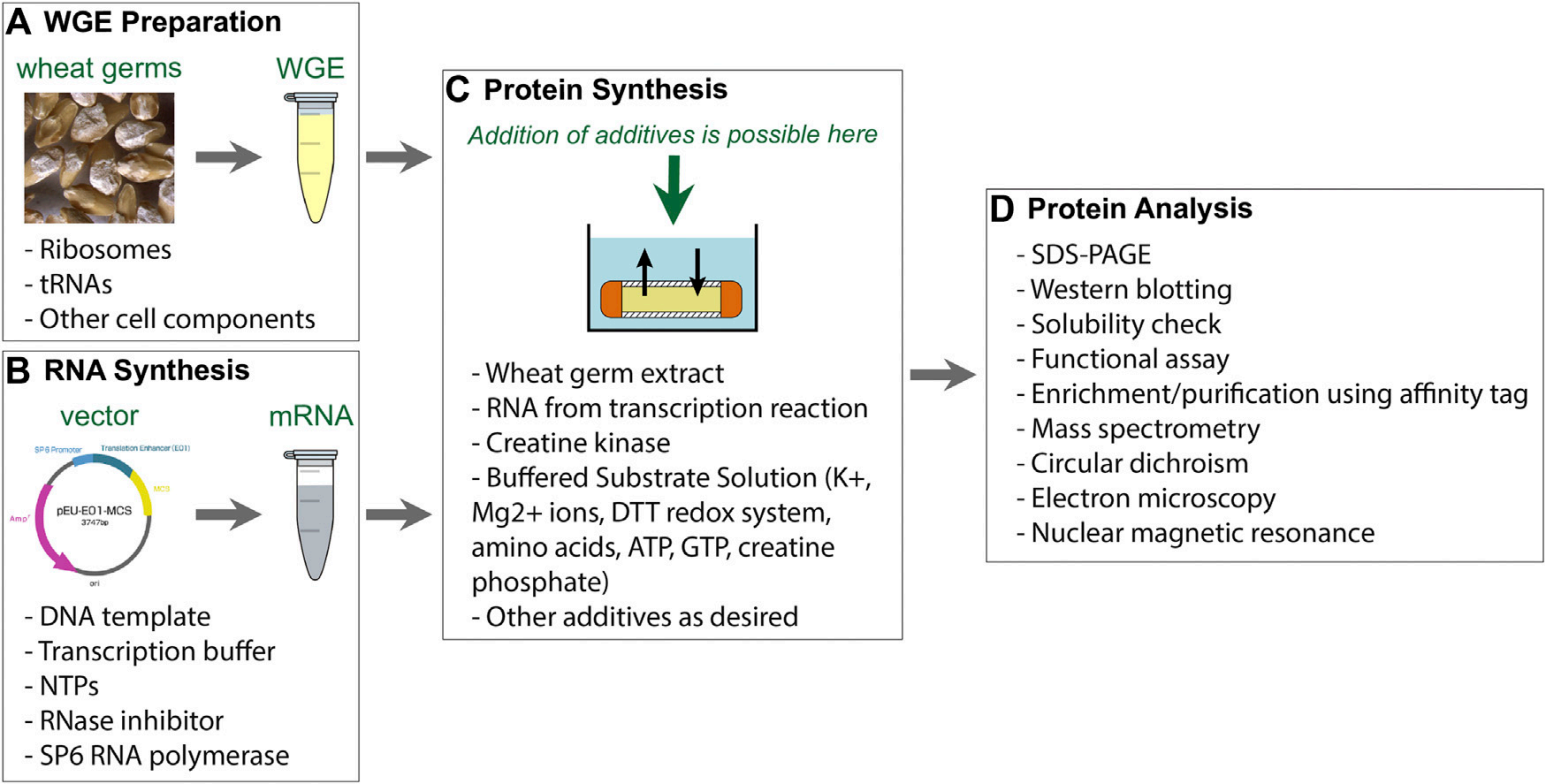
Components of WG-CFPS described by Takai et al. (2010). **(A)** Wheat germ extract (WGE) can be prepared from nontreated durum wheat. Alternatively, commercially available WGE can be used. **(B)** The WG-CFPS uses expression templates having a SP6 promoter to drive RNA synthesis and an E01 translational enhancer to induce cap-independent translation. The system can use circular and linear DNA templates to hold a cDNA encoding a protein. Alternatively, T7 RNA polymerase can be used as well under the same reaction conditions. The RNA is used as a template for protein synthesis. **(C)** The key components for protein synthesis are provided with the WGE. This includes the necessary ribosomes and tRNAs, but there are also other cell components in those extracts that may assist for example protein folding or possibly protein modification. Other key components are provided by the “Buffered Substrate Solution” which includes the amino acids, a DTT-based redox system, and a creatine kinase driving energy supply. Protein synthesis reactions can be modified as further explained in the text. **(D)** Protein synthesis can be confirmed by several different methods with the most commonly ones given in the figure.

Here, we review a typical workflow for using WG-CFPS and report our experiences about recombinantly preparing protein samples in this expression system. For all our experiments, we are using a WG-CFPS that had been developed in the Endo Lab at Ehime University (Sawasaki et al., 2002b). Endo and coworkers published the detailed protocol on how to prepare highly active wheat germ extracts by completely removing the endosperm in careful washing steps; the same protocol also describes how to utilize their wheat germ extracts in translation experiments (Takai et al., 2010). This protocol allows to establish extract preparation and CFPS in any reasonably equipped biochemistry laboratory; wheat germ extracts prepared according to the same procedure are also commercially available from CellFree Sciences (Japan). Figure 1 provides information on the basic steps for conducting protein expression experiments in this WG-CFPS. These conditions allow for direct expression of proteins in high-throughput experiments or also joint expression of several proteins in a single reaction, as shown, for example, for chromatin reconstruction experiments using premixed mRNAs for up to four core histones, three chromatin assembly factors, and histone H1 (Okimune et al., 2020). The expression of eight proteins in a single reaction is an impressive achievement not possible in most cell-based systems. However, the WG-CFPS can achieve this by simply adjusting the mRNA ratios in the translation reaction. The basic reaction conditions of the WG-CFPS can be adopted in many ways for more advanced applications further outlined in this review. The aim of this review is to give practical advice on how to plan and run such experiments and to highlight the extraordinary potential of the system, with a focus on (structural) studies on viral (membrane) proteins and the analysis of their assemblies.

## Experimental Design

**FIGURE 2:**
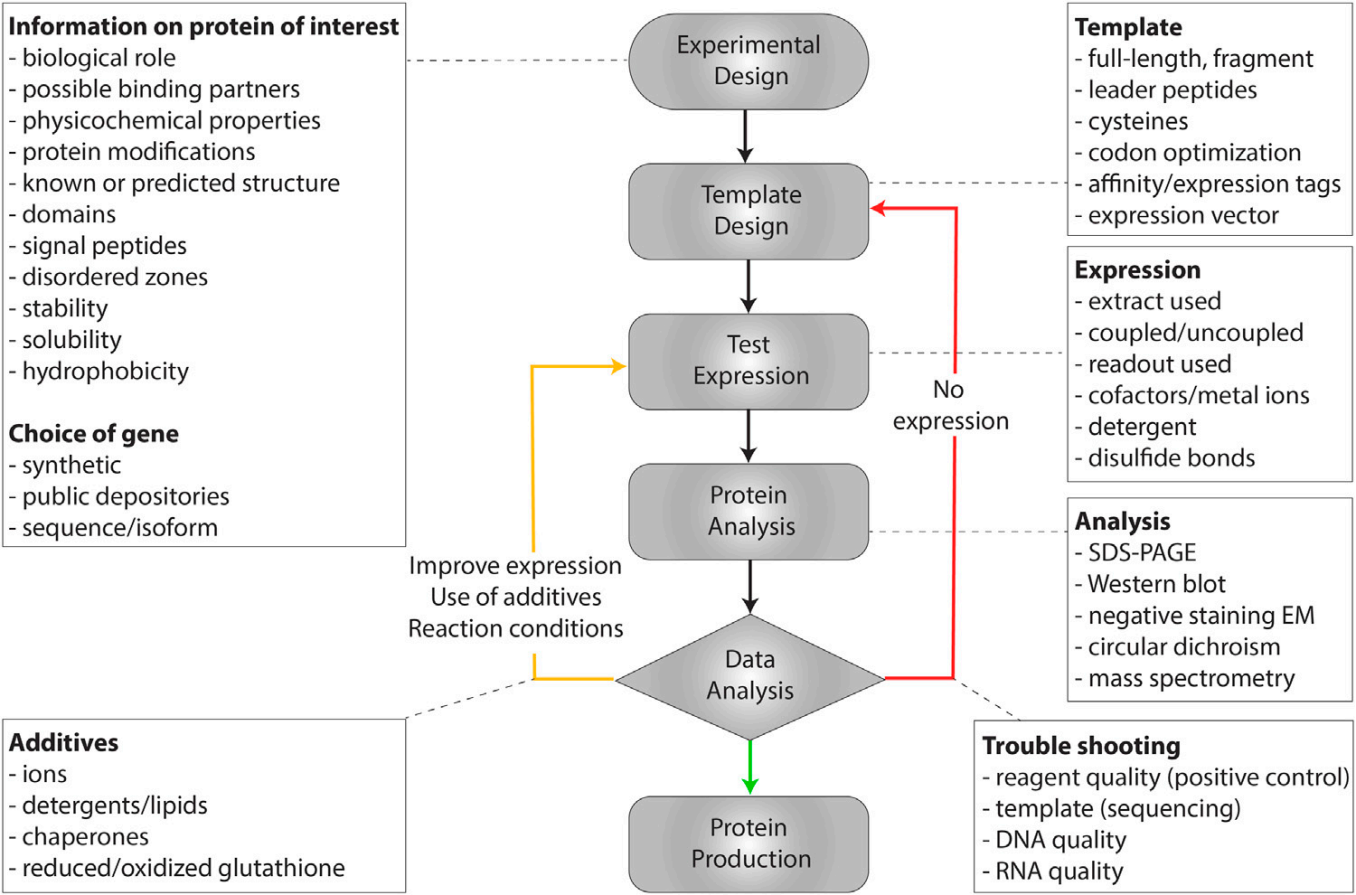
Workflow to establish protein synthesis in a WGS, with key points given at each step.

The workflow of an experiment in a wheat germ system (WGS) is given in Figure 2; refer to the guide by Wingfield (2015) for an overview on the purification of recombinant proteins which provides some general introduction into protein expression, particularly in *E. coli*. For experimental design, information on the nature of the protein is useful to better understand its requirements for production and purification. Next, the design of the cDNA template needed to produce the recombinant protein can include further considerations on working with fusion proteins such as constructs with affinity tags for detection and purification. The expression template determines conditions for the protein expression reactions and purification steps. For proteins requiring special conditions, the results of expression tests are analyzed and then optimized in iterative cycles. This may include different additives necessary to obtain soluble and correctly folded proteins. Finally, specific tests for samples later used for structural or functional analysis must be established to make sure that the protein is suitable for the intended use. This includes particularly biological and biophysical tests assessing whether the protein is correctly folded. We discuss these steps in the following sections in more detail.

## Information on the Protein of Interest

Before starting experiments, information about the protein of interest needs to be collected. This includes its biological role, possible binding partners, physicochemical properties, protein modifications, known or predicted structure, domains, signal peptides, disordered zones, stability, solubility, or hydrophobicity. This information enables optimal design of the expression template before a cDNA encoding the protein is cloned into a suitable expression vector or prepared by polymerase chain reaction (PCR) and is also useful for planning the expression reaction. For instance, the reaction may need to be performed at lower temperatures to change folding kinetics or decreasing hydrophobic interactions and self-aggregation. Currently, gene synthesis in combination with new cloning methods like Gibson assembly of overlapping DNA molecules (Gibson et al., 2009) offers a very flexible means to quickly prepare expression templates based on publicly available sequence information, including the results of high-speed sequencing experiments. However, one should be careful in selecting expression templates only based on assembled contigs from sequencing reads. It is best to use fully annotated protein/gene sequences if there is not a special reason for utilizing experimental sequence data. Since the protein sequence is the only needed information, gene synthesis may also be used to optimize the expression template by analysis of the RNA structure, looking for certain sequence elements, and to optimize the codon use for expression in a given host (Gustafsson et al., 2012; Athey et al., 2017) where codon optimization for expression in WG-CFPS is offered by most gene synthesis providers. Gene synthesis may further help with preparing templates for expression of fusion proteins having an affinity tag or creating artificial designer proteins such as protein standards for mass spectrometry (Takemori et al., 2017).

For many proteins, matching cDNAs have already been prepared during large-scale cloning projects (Harbers, 2008), and those clones can be obtained from public depositories or distributors. Particularly for human genes, large-scale cDNA collections are available including the ready-to-use open reading frame clones from the Human Gene and Protein Database (Goshima et al., 2008) or the international ORFeome Collaboration (Collaboration, 2016). For example, most of the cDNA clones in those two collections have been used in a study to identify reference peptides for targeted proteomics on the human proteome (Matsumoto et al., 2017). Since those clones are provided in Gateway entry vectors, the cDNA inserts can be easily transferred onto other vector formats (Reece-Hoyes and Walhout, 2018). Those and other cDNA collections can be readily searched for genes of interest as a convenient way to find cDNA clones from distributors rather than requesting published materials from other researchers or starting from scratch preparing them by gene synthesis. In general, for inquiries on a given gene, the “Gene” database at NCBI (https://www.ncbi.nlm.nih.gov/gene/) is a very good starting point (Brown et al., 2015; Coordinators, 2017). This database holds information on reference sequences from RefSeqs, maps, pathways, variations, phenotypes, and links to genome-, phenotype-, and locus-specific resources. The information provided in Gene can be valuable to learn more about a certain gene, while the sequence information may be useful for domain analysis, using gene synthesis services, or to confirm the sequence of a cDNA clone after an ID check. Moreover, Gene provides links to worldwide resources including cDNA clone providers (go to “Gene LinkOut,” you may have to click on the + sign to see the entire list at the end of the web page). NCBI allows suppliers to link (“LinkOut”) products and services on the specified gene shown in the Gene output page to help researchers to find resources in the public domain. This service is best known for links from publishers in PubMed but can also be used in other NCBI databases (https://www.ncbi.nlm.nih.gov/projects/linkout/). Refer to the following link on NCBI for more information on how to find cDNA clones in the public domain: https://www.ncbi.nlm.nih.gov/genome/clone/finding_cdna.shtml. In addition to the Gene database, there are many other protein-focused databases, like UniProt (https://www.uniprot.org/) that offers important information on the protein and its annotation, families, domains, and isoforms. For annotated proteins, the UniProt section on “amino acid modifications” includes possible disulfide bonds (Feige and Hendershot, 2018), which are formed under oxidizing conditions and thus may require changes to the protein expression and handling as further outlined below. Disulfide bonds are important for protein folding and stability and are mostly found in extracellular, secreted, and periplasmic proteins. We describe in the next chapter protein analysis tools available in the public domain that can provide information for template design beyond the information that is already provided in UniProt.

## Template Design

Template design is the starting point for making a protein, and a careful analysis of the protein and its features helps to prepare the template. There are several tools freely available on the Internet with information on protein properties, domain structures, or folding (refer to http://molbiol-tools.ca/Protein_Chemistry.htm and Table 1 for links to some of these tools).

**TABLE 1:** Protein analysis tools and selected databases.

Tool	Description	URL
Gene	Reference database and resources	https://www.ncbi.nlm.nih.gov/gene/
UniProt	Protein sequence and functional information	http://www.uniprot.org/
Wheat proteome	Reference on background protein analysis	https://www.wheatproteome.org/
Protein Chemistry	Links to useful tools	http://molbiol-tools.ca/Protein_Chemistry.htm
ProtParam tool	Calculating physical and chemical parameters	https://web.expasy.org/protparam/
Mfold	RNA folding	http://unafold.rna.albany.edu/?q=mfold
JPred	Protein secondary structure prediction	http://www.compbio.dundee.ac.uk/jpred/
Espript	Alignment and secondary structure prediction	http://espript.ibcp.fr/ESPript/ESPript/
Protter	Visualization of proteoforms	http://wlab.ethz.ch/protter/start/
Sable	Solvent accessibility	http://sable.cchmc.org
Scratch	Protein predictor on protein structures	http://scratch.proteomics.ics.uci.edu/
InterPro	Protein classification and predicting domains	https://www.ebi.ac.uk/interpro/
FFAS	Folding and function assignment	http://ffas.sanfordburnham.org/ffas-cgi/cgi/ffas.pl
CDTree	Protein domain hierarchy viewer and editor	https://www.ncbi.nlm.nih.gov/Structure/cdtree/cdtree.shtml
Cn3D	Macromolecular structure viewer	https://www.ncbi.nlm.nih.gov/Structure/CN3D/cn3d.shtml
VaProS	Variation effect on Protein structure and function	http://p4d-info.nig.ac.jp/vapros/
PSIPRED	Protein sequence analysis workbench	http://bioinf.cs.ucl.ac.uk/psipred/
PONDR	Predictor of natural disordered regions	http://www.pondr.com/
Protein data bank	3D structures of proteins	http://www.rcsb.org/pdb/home/home.do
PyMOL	Molecular visualization system	https://www.pymol.org/
TM finder	Transmembrane region finder	http://tmfinder.research.sickkids.ca/cgi-bin/TMFinderForm.cgi

**FIGURE 3:**
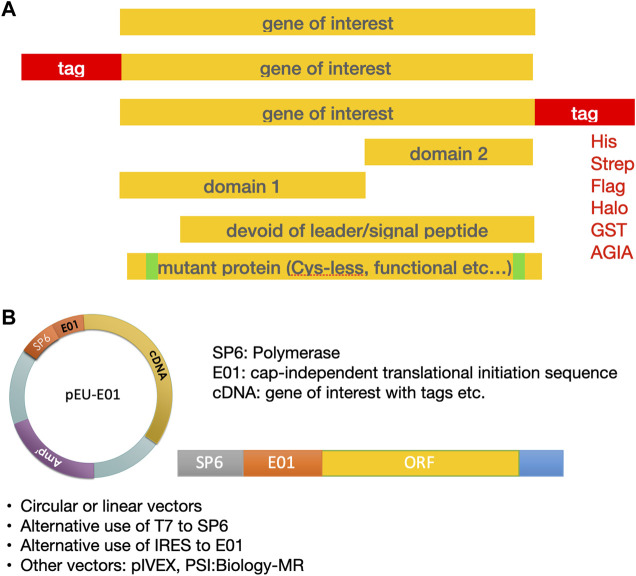
Template design for expression of the protein of interest. **(A)** Design of the gene sequence. **(B)** Both circular and linear DNA templates can be used for transcription.

While many proteins can be expressed in the WGS as full-length proteins in their native form, it may also be of interest to work on isolated domains or with other protein fragments (Figure 3). For example, some protein domains can reduce translation efficiency and may be removed from the recombinant proteins such as leader peptides, if not particularly needed for working with microsomes (Brodel et al., 2015). Leader peptides can be rather hydrophobic and frequently prevent correct folding of proteins. In general, the sequences at the N-terminus of proteins can have a large impact on protein yields in recombinant expression experiments. Therefore, it can be helpful to modify the N-terminus to improve yields for poorly expressed protein, for example, using a systematic tag variation strategy in combination with CFPS (Haberstock et al., 2012); a similar effect was described for N-terminal fusion with the GB1 domain (Michel and Wuthrich, 2012). Screening a library of 250,000 reporters led to the concept of a “short translational ramp” indicating that the amino acids in positions three and five impact protein yields (Verma et al., 2019).

Added sequences at the N- or C-terminus can have additional functions. The protein of interest may further be expressed with an affinity tag for later analysis and purification (see below). Other helpful tools such as Expasy ProtParam can help elucidate whether the primary sequence of the target protein is basic or acidic; which protein family it belongs to (e.g., UniProt; InterPro); whether it has hydrophobic stretches that need detergent or lipids to correctly fold (e.g., TM finder, for a more detailed review look at reference Punta et al., 2007); whether the protein has subdomains (e.g., Jpred, Scratch, and InterPro); whether it presents important functional motifs located directly at the N- or C-terminal, with which an affinity tag could interfere (e.g., FFAS; CDTree); which functions it could fulfill (e.g., UniProt); and what structures are predicted for folding (e.g., Protter). Corresponding websites for those tools are given in Table 1. Many of these tools are centralized on web portals, as, for example, on the Network Protein Sequence Analysis server (https://npsa-prabi.ibcp.fr/cgi-bin/npsa_automat.pl?page=/NPSA/npsa_server.html). Other important questions relate to the presence of cysteines and potential disulfide bonds in the protein (e.g., annotations in UniProt). Because of the importance of disulfide bonds for protein folding, stability, or complex formation, different computational methods have been described for working with them (Sun et al., 2017; Gao et al., 2020). However, we should point out that some cysteines do not form disulfide bonds in natively folded proteins. It can be important to prevent those cysteines from forming artificial disulfide bonds, since they can cause problems when proteins are refolding during later processing. Therefore, it may be better to mutate such cysteines to alanine or serine as done for the expression of the G protein-coupled neuropeptide Y receptor type 2, which had improved protein stability without any significant loss of functionality (Witte et al., 2013; Krug et al., 2020); refer to (Rawlings, 2018) for more information on membrane protein engineering. Another important example to note is zinc-binding motifs, which could require the use of additives like zinc ions or chaperones in the CFPS system.

For WG-CFPS, cDNA clones from various clone collections from different organisms (e.g., *Arabidopsis*, mouse, and human) have been used with good success, and it was commonly not necessary in those cases to do codon optimization. However, codon optimization is often used when the cDNA template was prepared by gene synthesis. This proved helpful when working on malaria-related projects, because *Plasmodium falciparum* uses very irregular A/T rich coding sequences (Arumugam et al., 2014). Codon optimization has been widely used, however, when expressing proteins in bacterial systems, which can have vastly different codon preferences as compared to higher organisms. It should be noted that codon usage has a direct influence on the elongation rate and thus regulates cotranslational folding (Yu et al., 2015). Theoretically, it could be useful to adjust the tRNA concentrations in a cell-free translation reaction to mimic those from the organism from which the recombinant protein is originally derived or to redesign the genetic code (Hibi et al., 2020). In principle, this could greatly assist correct protein folding; however, such experiments remain difficult without a good method to prepare individual tRNAs (Berg and Brandl, 2020) on a large scale. We expect more work in this area because studies on cancer genomes have revealed relevant synonymous mutations in tumors (Diederichs et al., 2016) that could function by changing protein folding via manipulating translation speed. Furthermore, it is interesting to note that structural limitations of tRNAs for binding to tRNA-modifying enzymes may have restricted the genetic code to 20 amino acids (Saint-Leger et al., 2016) and thus defined the chemical space for proteins. However, today, several approaches are in use to extend the genetic code and the space of amino acids that can be introduced into proteins (Arranz-Gibert et al., 2018). In addition to naturally occurring proteins, the WGS has also been successfully used to express artificial proteins such as the preparation of stable isotope-labeled peptide libraries (Takemori et al., 2016). Here, representative peptide sequences have been concatenated and expressed together in artificial proteins that were subjected to tryptic digestion to release individual peptides. This technique is a very cost-effective way to produce many different peptides when needed for quantitative protein mass spectrometry and proteome analysis.

## Use of Affinity Tags

When designing the expression template, considerations should also be given to means of protein detection, purification, or further modifications for the expression of fusion proteins. As mentioned above, fusion tags can have different functions including enhancing heterologous protein expression when placed at the N-terminus (Haberstock et al., 2012; Ki and Pack, 2020). This allowed, for instance, to increase yields of a GPCR 5–38 times, resulting in sufficient protein amounts for structural-functional studies (Lyukmanova et al., 2012b). Most fusion proteins have added sequences encoding an affinity tag that can be added at either end of the cDNA; small tags may also be added by primer extension PCR. Ready-to-use commercial expression vectors are available for the WGS or have been described in the literature (Bardoczy et al., 2008; Nagy et al., 2020). While affinity tags can be especially useful in protein purification, they also offer means for protein detection and analysis (Kimple et al., 2013; Wood, 2014; Yadav et al., 2016). Most affinity tags can be used in any protein expression system and are commonly host independent, but the recently developed AGIA-tag (Yano et al., 2016; Kido et al., 2020) and CP5-tag (Takeda et al., 2017) systems have presently only been described for the WG-CFPS. While epitope/antibody combinations allow for short tags and high-affinity binding, larger tags may add undesired functionalities to proteins of interest. It should also be noted that antibody-based tag systems are often better for analytical purposes, whereas they may be expensive for protein purification.

There is a preference for working with an affinity tag at the C-terminus to make sure that only full-length proteins are purified when translation is incomplete. Tags can be eliminated after purification by insertion of an enzymatic cleavage site for thrombin or Tobacco Etch Virus (TEV) proteases (Waugh, 2011). Examples of other cleavage sites are given in (Malhotra, 2009). Table 2 summarizes published affinity tags that have been used in combination with the WGS.

**TABLE 2:** Summary of tags whose use has been described for protein expression with the WGS.

Tag Examples of applications for WG-CFPS				Parameters to be considered for the choice of a tag	
Type	Main types of application	Position	References	Advantages	Drawbacks
Single tag					
His_6_	Translation setup and protein detection Purification by affinity chromatography	N-ter C-ter	(Malhotra, 2009; Vinarov et al., 2006a; Aoki et al., 2009; Revathi et al., 2010; Takahashi et al., 2012) (Aceti et al., 2015; Li et al., 2016; Novikova et al., 2018) (Fogeron et al., 2015a; Fogeron et al., 2015b; Li et al., 2016; Fogeron et al., 2017a; Minkoff et al., 2017)	Cost effective, easy, and fast purification process Affinity support endless reusable Small size tag with low impact on protein folding	WGE endogenous proteins bind to the affinity support Need for EDTA-free buffers
					
GST	Translation setup at small scale Purification by affinity chromatography	N-ter	(Malhotra, 2009; Vinarov et al., 2006a; Aoki et al., 2009; Revathi et al., 2010)	Solubility enhancement Low impact on protein folding when fused at the N-terminus Elution by enzymatic cleavage or competition Affinity support reusable up to five times	WGE endogenous proteins bind to the affinity support Higher purification cost than for His-tag Degradation of GST under reductive conditions
HaloTag	Pull-down assays Purification by affinity chromatography Protein detection by Western blot	N-ter	(Los et al., 2008; Bardoczy et al., 2008; Nagy et al., 2020)	Covalent bound, elution by enzymatic cleavage allowing for stringent washing conditions Higher binding capacity of the affinity support Highly specific interaction to the affinity support No binding of WGE endogenous proteins to the affinity support	Large size of the tag (34 kDa) Affinity support not reusable High purification cost
FLAG	Functional analysis Structural analysis by cryo-EM and crystallography Protein detection by Western blot Pull-down assays	N-ter	(Einhauer and Jungbauer, 2001; Bardoczy et al., 2008; Ramadan et al., 2015; Novikova et al., 2018; Nagy et al., 2020)	No binding of WGE endogenous proteins to the affinity support elution by enzymatic cleavage or by competition high protein recovery and high purity level in a only one-step purification process	Lower binding capacity of the affinity support Higher purification cost than for other tags
		C-ter	(Novikova et al., 2018)		
Strep-tag II	Purification by affinity chromatography	N-ter	(Schmidt and Scerra, 2007; David et al., 2019)	No binding of WGE endogenous proteins to the affinity support Cost-effective and easy purification process Elution by competition under native conditions Affinity support reusable up to five times	Slightly lower binding capacity of the affinity support than for His- and GST-tags Higher purification cost than for His- and GST-tags
		C-ter	(Fogeron et al., 2015a; Fogeron et al., 2015b; Li et al., 2016; Fogeron et al., 2017a; Minkoff et al., 2017; David et al., 2019)		
Dual tag					
Double-His_6_	Purification by affinity chromatography	N-ter	(Khan et al., 2006; Bardoczy et al., 2008; Nagy et al., 2020)	Improved binding capacity Increased detectability	
GST-His_6_	Pull-down assays Purification by affinity chromatography Protein detection by Western blot	N-ter/C-ter	(Bardoczy et al., 2008; Nagy et al., 2020)	Solubility enhancement through GST Efficient purification through GST Elimination of GST by TEV cleavage Highly sensitive detection through His-tag	
GST-AviTag	Pull-down assays Purification by affinity chromatography	N-ter/C-ter	(Cull and Schatz, 2000; Bardoczy et al., 2008; Nagy et al., 2020)	Solubility enhancement through GST Efficient purification through GST Elimination of GST by TEV cleavage Avitag allows for biotinylation of the protein	
His-Flag	*In vitro* binding assay (AlphaScreen)	N-ter	(Takahashi et al., 2012)	Efficient purification through His-tag Highly sensitive detection through FLAG-tag	
Flag-His	Structural analysis by cryo-EM and crystallography	N-ter/C-ter	(Novikova et al., 2018)	Two-step affinity purification for higher purity	
Twin-Strep-tag	Functional analysis Structural analysis by NMR	C-ter	(Schmidt et al., 2013; Fogeron et al., 2016; Boukadida et al., 2018; Jirasko et al., 2020)	Higher affinity than Strep-Tag	
His_6_-MBP	Purification by affinity chromatography	N-ter	(Aceti et al., 2015)	Solubility enhancement through MBP Efficient purification through His-tag	

GST, glutathione S-transferase; MBP, maltose-binding protein; WGE, wheat germ extract.

The Histidine tag or short His-tag (Malhotra, 2009), composed of 6 to 12 histidine residues, is very frequently used. When fused at either the N- or C-terminal end, it allows for a rapid, easy, and cost-effective purification on metal-chelating resins with high binding capacity. Nickel and cobalt resins are sensitive to reducing conditions such as those commonly used in CFPS reactions, and hence crude reaction mixtures should be diluted. Other commonly used purification tags like the glutathione S-transferase (GST) tag (Malhotra, 2009) are larger and can impart higher protein solubility when fused to the N-terminus (Malhotra, 2009). With a length of 220 amino acids (about 26 kDa in size), the GST-tag is large, which can be helpful in pull-down assays, where the GST protein not only facilitates the binding to a resin but also functions as a spacer to better expose the fused protein used in binding assays. The main drawback of the His-tag, and to a lesser extent of the GST-tag, is the unspecific binding of endogenous proteins from the wheat germ extract to metal-chelating and glutathione resins, which can lead to significant contamination of the affinity-purified proteins when working with proteins having low expression levels. This limitation can be addressed when using extracts pretreated on a nickel or glutathione resin for higher purity of His- or GST-tagged proteins (Takai et al., 2010; Harbers, 2014). Such extracts are commercially available (CellFree Sciences, Japan) for the His- and GST-tags. Both tags can also be combined with other tags where doubled-tagged proteins offer superior means to prepare highly purified proteins. It should further be mentioned that, against the His- and the GST-tag, commercial antibodies are available that can be used in protein detection, which is very handy to confirm protein expression when otherwise no antibodies are available recognizing the target protein.

As alternative approaches, the FLAG (Einhauer and Jungbauer, 2001) and Strep-tag II (Schmidt and Skerra, 2007) tags do often better remove background contaminations than possible for His- and GST-tagged proteins (Nagy et al., 2020). The FLAG-tag is an octapeptide (DYKDDDDK) that is recognized by a specific antibody which allows for sensitive protein detection. Three combined FLAG-tags were described for working with a WGS to achieve even higher binding affinity (Novikova et al., 2018). While a tagged protein can be eluted by competition with the peptide or by enterokinase cleavage, binding capacity of the resin is low, making affinity purification quite expensive and less attractive for large-scale routine protein production, but the FLAG-tag is an excellent tool for binding assays to study protein complexes. Here, the FLAG-tag has been used in PerkinElmer AlphaScreen assays (Nemoto et al., 2018) in combination with biotinylated proteins made in the WGS in the presence of added biotin and the biotin ligase BirA (Matsuoka et al., 2010). In contrast, the Strep-tag II (Schmidt and Skerra, 2007) allows for lower cost affinity purification, with high purity levels reached already after a single purification step. It uses a minimal peptide sequence (WSHPQFEK) with a high affinity to native streptavidin or an engineered streptavidin having even higher affinity for the tag (Strep-Tactin (Maertens et al., 2015)). Affinity purification using the Strep-tag II is rapid and easy to setup. The Strep-tag II is very suitable for routinely making proteins in the WGS. When even higher affinity is needed during purification, a Twin-Strep-tag (Schmidt et al., 2013) is available that can also be used with WGS (Fogeron et al., 2016; Boukadida et al., 2018; Jirasko et al., 2020).

The use of the HaloTag, a 297 amino acid peptide derived from a bacterial haloalkane dehalogenase (Los et al., 2008), has been recently described for the WGS (Nagy et al., 2020). Because the HaloTag forms a highly specific covalent bond with its synthetic ligand, this tag is of particular interest for pull-down assays, allowing for more stringent buffer conditions and washing steps (Los et al., 2008). Although affinity purification is possible, elution of proteins having a HaloTag must be performed by enzymatic cleavage (Nagy et al., 2020). This makes the method quite expensive and thus not very suitable for large-scale production.

In our hands, for NMR sample preparation, the Strep-tag II is so far the best choice since it combines high purity of the protein of interest with yields compatible with structural biology (Fogeron et al., 2015a; Fogeron et al., 2015b; Li et al., 2016; Fogeron et al., 2017a; Minkoff et al., 2017).

## Expression Templates

For routine protein expression, working with a dedicated expression vector is the best choice, although CFPS can also be done with linear DNA templates. Several vectors are available for use with the WGS, as outlined in (Bardoczy et al., 2008; Nagy et al., 2020) and references therein. We have always relied on vectors having the E01 enhancer (Kamura et al., 2005) to drive cap-independent translation (available from CellFree Sciences, Japan), but there are more expression vectors for WGS available from commercial providers (e.g., pIVEX Wheat Germ Vector Sets, biotechrabbit, Germany) and depositories (PSI:Biology-Materials Repository (PSI:Biology-MR)) sometimes using other initiation sites (Sawasaki et al., 2002b; Bardoczy et al., 2008). Commonly, the gene of interest should be inserted as near as possible to the E01 sequence to get better expression. For CFPS systems, the vectors commonly have promoters for an RNA polymerase like the SP6 or T7 RNA polymerases from bacteriophages, which catalyze the synthesis of RNA in a 5′–3′ direction (Mcallister and Raskin, 1993). In addition, a ribosomal binding site or translation enhancer is required to enable efficient protein expression. For the WGS, as well as for other eukaryotic systems, it is important to use a cap-independent translational initiation sequence like the E01 enhancer (Kamura et al., 2005) to avoid cumbersome steps for *in vitro* capping of the RNA transcripts. Alternatively, Internal Ribosome Entry Sites have been successfully used in various CFPS systems (Mikami et al., 2008; Anastasina et al., 2014; Hodgman and Jewett, 2014; Quast et al., 2016). This includes a Species Independent Translation Initiation Sequence that could be applied to prepare an expression vector for use in different CFPS systems, thus avoiding the need to clone into multiple expression vectors (Gagoski et al., 2015). Other translational enhancers in the 3′ untranslated region of the template have been described in the literature (Fan et al., 2012) that could potentially further improve protein expression (Ogawa et al., 2014). However, such elements are not commonly used in today’s expression systems. It was further reported that some noncoding antisense RNAs can stimulate the translation of a matching sense RNA. This observation led to developing synthetic long noncoding RNAs named SINEUPs to enhance protein translation *in vivo* or *in vitro* (Zucchelli et al., 2015a; Zucchelli et al., 2015b). To date, no examples for the successful use of this method in a WGS were published to our knowledge, although this biological principle may also exist in plants.

CFPS experiments can readily utilize linear DNA templates instead of circular vectors. While it is very convenient for many applications to directly prepare a template by the PCR, it should be noted that circular DNA templates are more stable and commonly provide better protein yields than linear DNA templates. Linear expression templates can be directly made by PCR methods (Schinn et al., 2016) without cloning experiments and thus allow for rapid expression screening. Different PCR protocols have been developed to add regularity sequences at the 5′ and 3′ ends of the coding region using overlap-extension PCR. In consecutive PCR reactions, a promoter to drive RNA expression and an enhancer to induce protein synthesis are added at the 5′ end; when working with the T7 RNA polymerase, a terminator sequence has to be added at the 3′ end. In addition to the regulatory sequences, the PCR primers can also be used to add short sequences encoding an affinity tag at either end. Caution is required when working with linear DNA in CFPS systems, because some extracts have an exonuclease activity that will damage or even entirely destroy a linear DNA template (Schinn et al., 2016). This problem was addressed by different approaches to protect or to extend the noncoding regions of the linear DNA templates. One elegant approach circularizes the PCR products before use in the expression reaction (Wu et al., 2007). However, the method uses the endogenous DNA ligase activity in *E. coli* S30 extracts, and only a quarter of the PCR products can be protected in this way. Other approaches to protect linear DNA templates have been described in the patent literature (Heindl et al., 2002). One easy-to-implement option is the use of biotinylated primers during PCR and later addition of streptavidin to the protein expression reaction to block exonucleases attacking the template from the ends. The same concept had been recently used when adding a DNA-binding protein to linear templates having matching binding sites at the ends (Zhu et al., 2020a). This approach had shown good template protection when working with an *E. coli* CFPS system, though it is less effective than circularizing the PCR product. The standard “Split-PCR” protocol commonly used in combination with the WGS uses an extended 3’ overhang to better protect the linear DNA templates (Sawasaki et al., 2002b). Uncoupling of transcription and translation reactions, as described below, may further help to avoid DNA degradation by exonuclease activities within the cell extracts used only in the translation reaction.

Regardless of the approach taken, we advise analyzing expression templates before use for having the correct sequence and all necessary elements for successful expression. It is our common routine to confirm the sequence of new expression vectors. We further recommend analysis of vector DNA on an agarose gel and determining the OD_260/280_ ratio to assure the purity of the DNA preparation. CFPS reactions are sensitive to the quality of DNA templates. If uncertain or unforeseen problems occur, often a phenol/chloroform extraction of the circular or even linear DNA templates can be immensely helpful to resolve problems with expression.

## Test Expression

Once the expression vectors or linear templates are available, all templates are then individually tested for expression of the target protein. Besides the templates, different wheat germ extracts can be compared for their properties foremost on the achieved protein yields. Although no clear data have been published, different extract preparations may lead to variations in posttranslational modifications during expression. Variations between extract preparations may be better controlled when using commercial reagents, with wheat germ extracts commercially available from different providers; alternatively, home-made wheat germ extracts can be used (Takai et al., 2010; Fogeron et al., 2015a).

The user of a cell-free translation reaction must choose between coupled or uncoupled reactions. In coupled reactions, transcription and translation are performed in a single reaction step, allowing for an easier setup and shorter overall time requirements. In uncoupled or linked reactions, the mRNA is prepared beforehand and then added to the wheat germ extract for the translation step. With modern protocols, the mRNA can be used directly after transcription without any prior purification (Takai et al., 2010). Although coupled reactions have been described for the WGS (Stueber et al., 1984), uncoupled reactions are usually preferred (Sawasaki et al., 2002a; Endo and Sawasaki, 2004, 2006; Takai et al., 2010). Uncoupling indeed allows for more flexibility to work under optimal reaction conditions (e.g., temperature), to use additives in the translation reaction without interfering with transcription, or to better identify and solve problems when they occur. These advantages clearly counterbalance the fact that uncoupled reactions might be more time-consuming. Note that both coupled and uncoupled reactions can be applied to the different reaction formats described in the Large-Scale Protein Production section.

**FIGURE 4:**
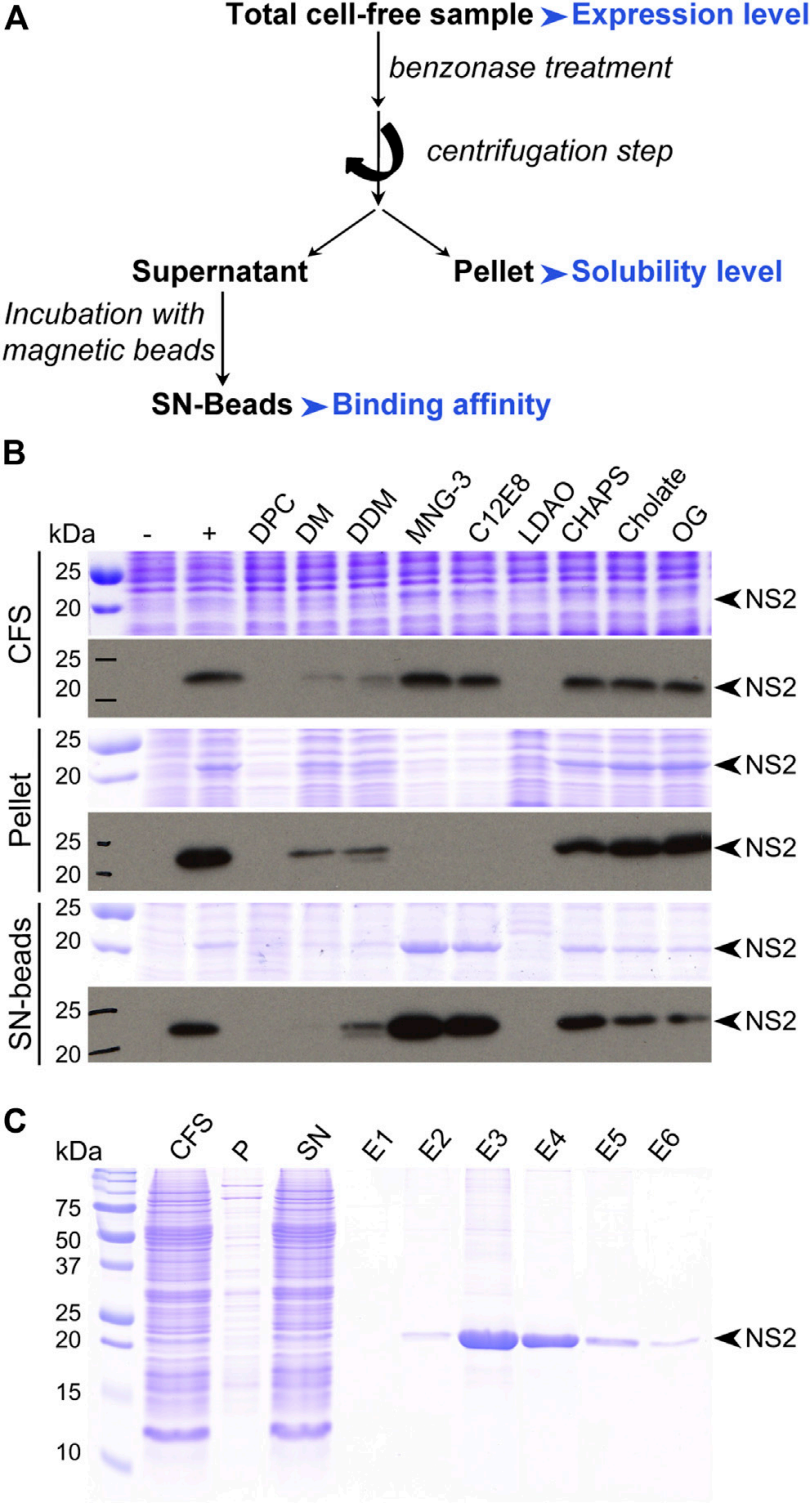
Protein analysis. **(A)** Typical flowchart for protein analysis after small-scale expression test. Parameters to be considered are highlighted in blue. **(B)** Small-scale expression test of the nonstructural protein 2 (NS2) from hepatitis C virus (HCV). This membrane protein was produced in the absence or presence of various detergents at a 0.1% concentration (w/v). Samples were analyzed by SDS-PAGE followed by Coomassie blue staining (upper panels) and Western blotting with an antibody against the Strep-tag II fused at the C-terminus of NS2 (lower panels). CFS, total cell-free sample; pellet, pellet obtained after centrifugation of CFS; SN-beads, supernatant obtained after centrifugation of CFS and incubated with Strep-Tactin magnetic beads to capture Strep-tag II-tagged NS2 protein; −, negative control (no NS2); +, positive control (NS2 expressed in the absence of detergent). The black arrowheads indicate NS2, adapted from Fogeron et al. (2015a). (C) SDS-PAGE analysis followed by Coomassie blue staining of the different steps from the affinity purification of the NS2 membrane protein produced directly in a solubilized form in the presence of MNG-3, adapted from Fogeron et al. (2015b).

Some proteins may require testing of different reaction conditions, which can be done in parallel, as shown in Figure 4 for added detergents. If there is an uncertainty on which regions of a protein could give best yields, PCR-based template generation can be used to test the expression of multiple protein fragments before cloning them into an expression vector (Novikova et al., 2018). Similarly, different affinity tags have been tested in this way to see their effect on protein expression (Haberstock et al., 2012; Kralicek, 2014).

Quick expression tests are preferably done in small batch reactions by adding a labeled-lysine-charged tRNA (FluoroTech™, Promega, United States) to expression reactions. The fluorescently labeled lysine is randomly incorporated at AAA codons into the synthesized protein during the translation reaction, thus allowing for easy background-free detection of proteins (Zhao et al., 2010; Novikova et al., 2018). After completion of the translation reaction, the labeled protein can be directly detected by SDS-PAGE using a laser-based fluorescent gel scanner; we recommend digesting the remaining labeled tRNA by RNase A treatment before loading onto the gel. In an optimized expression system, only the newly synthesized protein from the added template should be visible on SDS-PAGE as there is no background expression in the WGS. While the labeling reaction is providing information on whether the protein can be made from an expression template, it is good to also perform a regular cell-free protein expression experiment without the fluorescent label to further test protein yields, solubility, purification methods, and possibly protein function (Fogeron et al., 2017a), as it is unclear whether in certain cases the randomly incorporated labeled lysine could interfere with protein functions. The expression test experiments should be further extended if the protein of interest requires disulfide bonds, certain cofactors, the addition of metal ions or is, for example, a membrane protein with expected low solubility. We will provide below more information on additives that could be tested to improve protein expression and quality.

## Protein Analysis

SDS-PAGE analysis effectively assesses the expression and solubility of the protein within translation reactions, where it can be helpful to compare to a negative control expression reaction lacking the template or using an empty expression vector. While staining the proteins in the gel might be sufficient for protein detection, it can be advantageous to detect the protein of interest by Western blotting using a suitable antibody, which can also be directed against an affinity tag. Treating samples with benzonase, an endonuclease degrading DNA and RNA independently of their shape allows removing nucleic acids from the translation reaction. As indicated in Figure 4, both the full reaction mixture, the supernatant and pellet after centrifugation of the crude reaction mixture (e.g., at 20,000 g for 30 min) are analyzed to assess protein expression and solubility. The protein in the supernatant fraction can, for better visibility on the gel, be enriched using magnetic beads which can capture a tagged protein via the tag. Magnetic beads are fast, easy, and very convenient to use; they offer a higher binding capacity than standard chromatography resins and allow for efficient automation. Another fraction to be analyzed is the remaining supernatant of the binding assay, to confirm that the tag had worked properly.

SDS-PAGE of the full reaction already reveals if synthesis was successful. The protein in this fraction can best be seen by Western blotting, since there are many contaminating proteins present in the crude reaction mixture. When insoluble, the pellet fraction will be enriched in the protein target, which can typically be the case for membrane proteins or nucleic-acid-binding proteins like transcription factors. If this is the case, the protein can sometimes be seen using Coomassie staining, since there are few insoluble proteins present in wheat germ extracts. Otherwise, it should be identified using Western blotting for more reliable detection. The soluble fraction concentrated on beads will show the protein when soluble and if it attaches correctly to the beads via its tag. If the tag is inaccessible, the protein will remain in the soluble fraction. Confirming binding of the tag can help design the subsequent purification steps. Most structure determination techniques require soluble proteins; still, solid-state NMR and cryo-EM can be applied to proteins which are localized to the pellet fraction, due to either their size or aggregation state. One should however mention that while pellets formed by autoassembling proteins, or RNA-interacting proteins, can be correctly folded, membrane proteins found in the pellet after expression in absence of a detergent are likely misfolded. In the latter case, solubilization is an asset, as membrane reconstitution can then be done subsequently. Soluble expression can often be induced by additives (see below); data analysis will be carried out in a similar way to assess protein synthesis, solubility, and binding to the magnetic beads as proxy for purification. In structural genomics studies, at this point, one can distinguish if a protein will be directed to analyses using a soluble protein, like solution-state NMR or X-ray crystallography, or if it will need approaches that can target insoluble proteins, as solid-state NMR and cryo-EM.

SDS-PAGE also allows confirming expected protein size, as well as stability, and the presence of degradation products (which should be largely absent, since wheat germ extracts have no significant protease activity). If a protein is expressed in a soluble state and attaches to the magnetic beads, one can proceed to column purification via standard protocols for the used tag, and fractions can be analyzed using SDS-PAGE. The purified protein can be used for the first biophysical characterization using mass spectrometry, which allows confirming its identity, as well as detecting possible posttranslation modifications. The latter, typically phosphorylation, was shown to be possible in the WGS and took place on sites identified *in vivo* (David et al., 2019). Acetylation was also observed (unpublished). An enzymatic test on the translation reaction can often determine already whether the protein is functional or not; precautions must be taken on background activities in the wheat germ extract, however. Also, when autoassemblies are expected to form, electron microscopy analysis allows for their direct observation, often also in the crude reaction (David et al., 2018; Wang et al., 2019). In addition, the secondary structure of the protein of interest can be investigated by circular dichroism (Kelly et al., 2005).

## Troubleshooting Poor Expression

When no expressed protein can be detected, the reaction conditions and template design should be checked for possible errors. Negative results are often about poor detection or the inability to see the overexpressed protein over the background of proteins from the extract. Also, often proteins may not show on SDS-PAGE at the expected molecular weight. Both problems can be addressed by Western blotting. Using the FluoroTect™ labeling method described above, we have seen only very few cases where no protein could be detected after expression in the WGS, as the method is very sensitive (note that the free label will run at the front of the gel which may interfere with very small proteins and thus may be better removed before SDS-PAGE analysis).

Further troubleshooting should consider the following points working with uncoupled reactions to better understand potential problems during transcription and translation: 1. Confirm the expression template was made correctly and no mistakes have been made during template design and preparation. 2. Confirm the DNA quality of the template on an agarose gel and measuring the OD_260/280_. 3. Confirm the RNA quality using agarose gel or capillary electrophoresis; CFPS reactions must be done under RNase-free conditions. 4. Confirm reagent quality by working with a positive control known to work well in the expression system.

Regarding the template design, an N-terminal tag can have an impact on the secondary structure of RNA, and thus on protein expression where, for example, hairpin loops tend to repress translation. RNA secondary structures can be analyzed using the Mfold software (Zuker, 2003) (http://unafold.rna.albany.edu/?q=mfold/RNA-Folding-Form). In case of low protein yields, changes to the N-terminus could also be considered as, for example, shown for making Growth Hormone Secretagogue Receptor in a CFPS (Pacull et al., 2020). Further, for optimal purity of the DNA template, a phenol/chloroform extraction is recommended. A sign for an efficient transcription reaction is the appearance of a white magnesium pyrophosphate precipitate. Agarose gel electrophoresis allows verifying the expected size of the RNA. When working with circular DNA and an SP6 promotor, the RNA can form a ladder as the polymerase may run several times around the vector. While wheat germ extracts can be stored for an extended time at −80°C, they are overly sensitive to freeze/thawing cycles, or any storage at higher temperatures. We advise using a positive control like, for example, expressing an easy-to-detect Green Fluorescent Protein (GFP) to confirm the performance of wheat germ extracts and other reagents. Moreover, commercial buffers are preferred since they are less error prone.

## Optimizing Expression Reaction Conditions

### Expression Conditions

The expression yield is an important parameter, especially for structural studies which require higher amounts of protein. The temperature during translation reactions can have an impact on both expression yield and protein folding. When protein yields are not satisfying, it is thus worth testing different temperatures for protein synthesis within a range from 4 to 25°C; wheat germ extracts lose activity above 25°C. The lower the temperature is, the longer the translation reaction must be.

### Additives

*In vitro* reactions allow for the addition of factors that may be required for optimal protein folding, function, or solubility. Most common examples are the addition of isotope-labeled amino acids (Makino et al., 2010), metal ions like zinc (Okada et al., 2009) and iron (Samuel et al., 2015), redox reagents supporting the formation of disulfide bonds (Saaranen and Ruddock, 2019), or detergents and lipids (Sachse et al., 2014). Additionally, chaperones may be used to support or modulate folding. However, one should test beforehand whether additives do not interfere with protein synthesis. Note that wheat germ extracts commonly contain some lipids and metal cofactors that may assist already protein expression (Goren et al., 2009).

Additives, such as detergents, lipids, or chaperones may also help to improve protein yields. We have experienced that adding detergent to protein synthesis could improve their expression level and purity.

Ions

Besides zinc and iron ions, other ions can be used in the WGS. Refer to Table 3 for different ions that had been tested for use in the WGS (unpublished data provided by F. Tanabe and R. Morishita). In the table, we give the highest ion concentrations that can be used in the translation reactions without inhibiting synthesis in separated transcription and translation reactions. Goren and Fox (Goren and Fox, 2008) described the preparation of a functional human stearoyl-CoA desaturase complex by coexpression in the WGS, which requires nonheme iron for its catalytic function. Because the wheat germ extract lacked the necessary amount of iron ions and heme, ascorbate stabilized Fe^2+^ was subsequently added to their proteoliposome preparation to activate the complex. They also provide information on an elemental analysis of a wheat germ extract. As another example, the yeast (m^2^G10) methyltransferase (a Trm11 and Trm112 complex) was prepared using the WGS for coexpression and complex formation (Okada et al., 2009). Since Trm112 contains two zinc fingers, the authors showed that the system could be used in the presence of up to 20 µM added ZnCl_2_ without reducing protein yields.

Detergents and lipids

**TABLE 3:** Maximal concentration for added ions.

Ion	Salt	Maximal concentration (µM)
Mn^2+^	Chloride	100
	Acetate	100
Mg^2+a^	Chloride	100
	Acetate	100
Ca^2+^	Chloride	100
	Acetate	100
Cu^2+^	Chloride	100
	Acetate	100
Cd^2+^	Chloride	100
	Acetate	100
Co^2+^	Chloride	100
	Acetate	100
Fe^2+b^	Chloride	10
Ni^2+^	Chloride	100
	Acetate	100
Zn^2+^	Chloride	10
	Acetate	100

Data obtained for expression of GFP using wheat germ extract WEPRO7240 (CellFree Sciences, Japan) on a bilayer format.

^a^CFPS systems are critically dependent on the Mg concentration.

^b^About 25% reduction of protein yield when using 100 µM ferrous chloride.

Detergents and lipids are of special interest for working with membrane proteins, which are today the most important drug targets for therapy (Hopkins and Groom, 2002; Arinaminpathy et al., 2009). However, membrane proteins are notoriously difficult to express in living cells since they are often toxic and may depend on the lipid composition of membranes (Harayama and Riezman, 2018). This makes CFPS a highly valuable alternative, where the use of CFPS systems for the preparation of G protein-coupled receptors for structural investigations was recently reviewed (Kögler et al., 2019). Three dedicated protocols were established for their expression in CFPS systems: 1) the precipitate mode, 2) working in the presence of detergents, or 3) working in the presence of lipids. In the first mode, protein precipitates form during synthesis and can be afterward efficiently solubilized with a detergent (Klammt et al., 2004). Although there is evidence that detergent solubilization of membrane protein precipitates produced in the *E. coli* CFPS systems could result in functionally folded proteins (Klammt et al., 2004; Sansuk et al., 2008), it was also shown that such a process could lead to inactive proteins (Klammt et al., 2005; Klammt et al., 2007). Examples for solubilization and refolding after expression in *E. coli* CFPS have been published for the Growth Hormone Secretagogue Receptor (Pacull et al., 2020) and Neuropeptide Y2 Receptor (Krug et al., 2020). As lipids are not fully removed during wheat germ extract preparation, they may bind to proteins (Schwarz et al., 2008), which could explain why membrane proteins expressed in the precipitate mode are sometimes partially soluble.

An interesting alternative is the production of membrane proteins in the presence of detergents. While ionic detergents often denature proteins, nonionic and zwitterionic detergents are mild for membrane protein solubilization and in many cases preserve protein folding. Above the critical micelle concentration (CMC), detergents in aqueous solutions spontaneously form micellar structures (Garavito and Ferguson-Miller, 2001; Seddon et al., 2004). The CMC is influenced by pH, ionic strength, temperature, and the presence of protein, lipid, and other detergent molecules. Membrane protein expression in the presence of detergent leads to the formation of proteomicelles. Detergents are available instantly at the ribosomes, eliminating problems encountered regarding the transport to membranes and translocation processes of synthesized proteins (Schwarz et al., 2008). Importantly, not all detergents are compatible with CFPS systems. Their use in *E. coli* lysates has been broadly reported (Berrier et al., 2004; Elbaz et al., 2004; Ishihara et al., 2005; Klammt et al., 2005; Schwarz et al., 2008; Deniaud et al., 2010; Miot and Betton, 2011), suggesting that mild detergents with low CMC values allow for optimal solubilization yields without interfering with expression yields. However, some detergents affected protein expression levels in the WGS (Genji et al., 2010). Table 4 summarizes detergents whose use was described for the WGS. Detergent concentration can also impact both protein expression and solubilization levels. Alternatives to traditional detergents, such as the linear carbohydrate-based polymer NVoy (Guild et al., 2011) and peptide surfactants (Periasamy et al., 2013), have been described for use in the WGS as well. In addition, the use of fluorinated compounds (Park et al., 2007; Park et al., 2011; Blesneac et al., 2012) and amphipols (Popot, 2010) has been reported for *E. coli* based systems and supports direct membrane protein reconstitution into membranes (Nagy et al., 2001; Park et al., 2007). Note however that there is only one commercially available amphipol compatible with CFPS (NAPol) (Popot, 2010; Park et al., 2011). To be analyzed in a native-like environment, membrane proteins expressed in the presence of traditional detergents or alternative surfactants can be reconstituted in lipids after purification (Seddon et al., 2004; Fogeron et al., 2016; Lacabanne et al., 2017; Jirasko et al., 2020), which has been described as the most successful approach for membrane protein insertion into membranes (Bayburt and Sligar, 2010). As protein loss needs to be minimized, fast lipid reconstitution without the need for extensive protein handling is an asset. This is possible by using, instead of lengthy dialysis for detergent removal (Althoff et al., 2012; Lacabanne et al., 2017), complexation of the detergents with cyclodextrin (Degrip et al., 1998). Proteoliposomes can then simply be separated by centrifugation for further analysis (Jirasko et al., 2020).

**TABLE 4:** Summary of detergents whose use has been described to produce membrane proteins with the WGS.

Detergents		Proteins	Yield	Solubility	References	Applications/comments
*Anionic*						
Cholate	3,7,12-Trihydroxy-5-cholan-24-oic acid	AtPPT1	▽		(Nozawa et al., 2007)	
		Insect odorant receptor subunits		–	(Carraher et al., 2013)	
		Nonstructural proteins from HCV	▽		(Fogeron et al., 2015a)	Protein expression
Deoxycholate	3,12-Dihydroxy-5-cholan-24-oic acid	Insect odorant receptor subunits		–	(Carraher et al., 2013)	
N-Lauryl sarcosine		Insect odorant receptor subunits		▲	(Carraher et al., 2013)	Not suitable for functional or structural studies
SDS	Sodium dodecyl sulfate	Insect odorant receptor subunits		▲	(Carraher et al., 2013)	Not suitable for functional or structural studies
*Zwitterionic*						
CHAPS	Steroid derivative (3-((3-cholamidopropyl)dimethylammonio)-1-propansulfonat)	AtPPT1	▽		(Nozawa et al., 2007)	
		Bacteriorhodopsin	▽		(Genji et al., 2010)	
		CrdS (*Agrobacterium* curdlan synthase)	▽		(Periasamy et al., 2013)	Protein expression
		Nonstructural proteins from HCV	▽		(Fogeron et al., 2015a)	
		CD36	▽	▲	CFS	
		EDG3; GPR84	▽		CFS	
DPC	Monochain phosphocoline (dodecyl-phosphocholine)	Nonstructural proteins from HCV	▽		(Fogeron et al., 2015a)	Protein expression
LDAO	Lauryl dimethyl amide oxide	Nonstructural proteins from HCV	▽		(Fogeron et al., 2015a)	Protein expression
LPPG	1-Palmitoyl-2-hydroxy-sn-glycero-3-[phospho-rac-(1-glycerol)]	F2R, CDC91L1, EDG3, PINK1, CCR7, GPR84		▲	CFS	
Zwittergent 3–16		Insect odorant receptor subunits		▲	(Carraher et al., 2013)	Insertion of purified proteins into preformed liposomes
*Nonionic*						
Brij-35	Polyoxyethylene alkyl-ether	AtPPT1		▲	(Nozawa et al., 2007)	
		Bacteriorhodopsin		▲	(Goren and Fox, 2008)	
		hSCD1 (human stearoyl-CoA desaturase)		▲	(Goren and Fox, 2008)	
		Insect odorant receptor subunits		▲	(Carraher et al., 2013)	Insertion of purified proteins into preformed liposomes
		F2R, CDC91L1, CD36, PINK1, CCR7, and GPR84		▲	CFS	
		EDG3	▲	▲	CFS	
Brij-58	Polyoxyethylene alkyl-ether	AtPPT1		▲	(Nozawa et al., 2007)	
		Olfactory receptors (GPCRs)	▽	▲	(Kaiser et al., 2008)	
		Bacteriorhodopsin		–	(Genji et al., 2010)	Protein not functional
		CrdS (*Agrobacterium* curdlan synthase)		▲	(Periasamy et al., 2013)	Insertion of purified protein into nanodiscs
		Insect odorant receptor subunits		▲	(Carraher et al., 2013)	Insertion of purified proteins into preformed liposomes
		F2R, CDC91L1, CD36, PINK1, CCR7, and GPR84		▲	CFS	
		EDG3	▲	▲	CFS	
Brij-78	Polyoxyethylene alkyl-ether	AtPPT1		▲	(Nozawa et al., 2007)	
		Insect odorant receptor subunits		▲	(Carraher et al., 2013)	Insertion of purified proteins into preformed liposomes
Brij-97	Polyoxyethylene alkyl-ether	AtPPT1		▲	(Nozawa et al., 2007)	
Brij-98	Polyoxyethylene alkyl-ether	AtPPT1		▲	(Nozawa et al., 2007)	
		Insect odorant receptor subunits		▲	(Carraher et al., 2013)	Insertion of purified proteins into preformed liposomes
C12E8	Dodecyl octaethylene glycol ether	Nonstructural proteins from HCV		▲	(Fogeron et al., 2015a)	Protein expression
Digitonin	Steroid derivative	AtPPT1	▽	▲	(Nozawa et al., 2007)	
		Olfactory receptors (GPCRs)		▲	(Kaiser et al., 2008)	
		Bacteriorhodopsin		–	(Genji et al., 2010)	Protein not functional
		CrdS (*Agrobacterium* curdlan synthase)		▲	(Periasamy et al., 2013)	
		Insect odorant receptor subunits		▲	(Carraher et al., 2013)	Insertion of purified proteins into preformed liposomes
		F2R, CDC91L1, CD36, EDG3, PINK1, CCR7, and GPR84	▲	▲	CFS	
DM	Alkyl glucoside (n-decyl-D-maltoside)	Bacteriorhodopsin	▽	▲	(Chae et al., 2010a)	
		Nonstructural proteins from HCV	▽		(Fogeron et al., 2015a)	Protein expression
DDM	Alkyl glucoside (n-dodecyl-D-maltoside)	AtPPT1	▽		(Nozawa et al., 2007)	
		Bacteriorhodopsin	▽	▲	(Chae et al., 2010a)	
		hVDAC1	▽	▲	(Nozawa et al., 2007)	Protein crystallization
		AAC (ADP/ATP carrier)	▽		(Long et al., 2012)	
		Insect odorant receptor subunits		–	(Carraher et al., 2013)	Insertion of purified proteins into preformed liposomes
		Nonstructural proteins from HCV	▽	▲	(Fogeron et al., 2015a)	Protein expression
		F2R	▽	▲	CFS	
MNG-3	Lauryl maltose neopentyl glycol	Bacteriorhodopsin		▲	(Chae et al., 2010b)	
		Nonstructural proteins from HCV		▲		Structural analysis by NMR
						Functional analysis
		Envelope proteins from duck hepatitis B virus		▲	(David et al., 2018; David et al., 2019)	
Nonidet P-40	Polyethylene glycol derivative	AtPPT1	▽	▲	(Nozawa et al., 2007)	
		CrdS (*Agrobacterium* curdlan synthase)	▽		(Periasamy et al., 2013)	
		CDC91L1	▽		CFS	
		CD36, EDG3, and PINK1		▲	CFS	
		CCR7 and GPR84	▽	▲	CFS	
β-OG	Alkyl glucoside (n-octyl-d-glucopyranoside)	Insect odorant receptor subunits		–	(Carraher et al., 2013)	
		CrdS (*Agrobacterium* curdlan synthase)	▽		(Periasamy et al., 2013)	
		Nonstructural proteins from HCV	▽		(Fogeron et al., 2015a)	Protein expression
		EWSR1, CDC91L1, CD36, PINK1, and GPR84	▽		CFS	
Triton X-100	Polyethylene glycol derivative	AtPPT1	▽	▲	(Nozawa et al., 2007)	
		CrdS (*Agrobacterium* curdlan synthase)		▲	(Periasamy et al., 2013)	
		Insect odorant receptor subunits		▲	(Carraher et al., 2013)	Insertion of purified proteins into preformed liposomes
		F2R	▽	▲	CFS	
		CDC91L1, CD36, EDG3, PINK1, CCR7, and GPR84		▲	CFS	
Triton X-114	Polyethylene glycol derivative	Insect odorant receptor subunits		▲	(Carraher et al., 2013)	Insertion of purified proteins into preformed liposomes
Tween-20	Polyoxyethylene alkyl-ether	AtPPT1	▽		(Nozawa et al., 2007)	
		F2R, CD36, PINK1, and CCR7	▽	▲	CFS	
		EWSR1, CDC91L1, and GPR84	▽		CFS	
		EDG3		▲	CFS	
Tween-40	Polyoxyethylene alkyl-ether	AtPPT1		▲	(Nozawa et al., 2007)	
Tween-60	Polyoxyethylene alkyl-ether	AtPPT1		▲	(Nozawa et al., 2007)	
Tween-80	Polyoxyethylene alkyl-ether	AtPPT1		▲	(Nozawa et al., 2007)	
		CrdS (*Agrobacterium* curdlan synthase)		▲	(Periasamy et al., 2013)	
*Mixtures*						
Fos-choline	FC-12 or FC-14	Bacteriorhodopsin		▲	(Genji et al., 2010; Nozawa and Tozawa, 2014b)	Counteracting the inhibitory effect of detergent Expression and purification of functional protein
CHAPS						

▽, decrease in yield or solubility.

▲, increase in yield or solubility.

–, no effect on solubility level.

HCV, hepatitis C virus. CFS, CellFree Sciences (M. Denda et al., poster presentation at PepTalk 2011).

Another option is to express the proteins in the presence of lipids, as membrane proteins can cotranslationally incorporate into the lipid bilayer to form directly proteoliposomes (Nozawa et al., 2007). Different protocols have been developed in this context (Shadiac et al., 2013; Zhou and Takeda, 2020). Although the endoplasmic reticulum (ER) is removed during wheat germ extract preparation, mimicking the natural membrane environment is indeed possible with the addition of lipids to the translation reaction. CFPS systems tolerate relatively high concentrations of lipids and lipid mixtures, and even slightly beneficial effects have been observed on the expression efficiency (Klammt et al., 2004). Most often lipids are used in the WGS in the form of liposomes (Akbarzadeh et al., 2013), which are artificial spherical vesicles formed by lipid bilayers from either synthetic lipids or biological lipid extracts (Akbarzadeh et al., 2013). Insertion of membrane proteins into liposomes leads to the direct formation of proteoliposomes which are generally isolated by ultracentrifugation on density gradients (Nozawa et al., 2011; Periasamy et al., 2013). Such proteoliposomes can be easily purified and have been used in studies on membrane proteins (Banerjee and Datta, 1983; Rigaud, 2002; Wang and Tonggu, 2015). Lipid type and composition are highly important to ensure cotranslational insertion (Periasamy et al., 2013), and screening biologically relevant lipids instead of using commercially available lipids might be a better choice. Examples of membrane proteins produced in the presence of liposomes using a WGS are summarized in Table 5. This approach is in theory very attractive, but not all proteins can be integrated into liposomes, some requiring a more complex lipid environment and others depending on the translocon machinery (Sachse et al., 2014). The absence of the translocon can, however, be problematic for the topology of multispanning membrane proteins. Also, when low lipid-to-protein ratios are crucial, it might be that these ratios cannot be reached using lipid addition, since spontaneous insertion might not be quantitative.

**TABLE 5:** Summary of lipids whose use has been described for the production of membrane proteins with the WGS.

Lipid composition	Proteins	References	Applications/comments

*Anionic lipids*			
DOPG	CrdS (*Agrobacterium* curdlan synthase)	(Periasamy et al., 2013)	No protein expression
*Cationic lipids*			
DOTAP	CrdS (*Agrobacterium* curdlan synthase)	(Periasamy et al., 2013)	No protein expression
*Zwitterionic lipids*			
Asolectin	AtPPT1 (*Arabidopsis thaliana* phosphoenolpyruvate/phosphate translocator 1) and 40 other membrane proteins	(Nozawa et al., 2011; Periasamy et al., 2013)	Functional analysis
			Isolation on density gradient
	AtDTC (*Arabidopsis thaliana* dicarboxylate/tricarboxylate carrier)	(Nozawa and Tozawa, 2014b)	
	PfDTC (*Plasmodium falciparum* dicarboxylate-tricarboxylate carrier homolog)	(Nozawa et al., 2011; Periasamy et al., 2013)	Transport activity
	CrdS (*Agrobacterium* curdlan synthase)	(Periasamy et al., 2013)	
	Human dopamine D_1_ receptors	(Arimitsu et al., 2014)	Receptor binding activity
	Ant1p (*Saccharomyces cerevisiae* adenine nucleotide transporter)	(Nozawa and Tozawa, 2014a)	Transport activity of ATP/AMP exchange
DMPC	Bacteriorhodopsin	(Genji et al., 2010)	Bacteriorhodopsin not functional
	CrdS (*Agrobacterium* curdlan synthase)	(Periasamy et al., 2013)	
DOPC	Cytochrome b_5_	(Nomura et al., 2008)	Transport activity
	CrdS (*Agrobacterium* curdlan synthase)	(Periasamy et al., 2013)	
EYPC	Cytochrome b_5_	(Nomura et al., 2008)	Transport activity
POPC	CrdS (*Agrobacterium* curdlan synthase)	(Periasamy et al., 2013)	
POPE	CrdS (*Agrobacterium* curdlan synthase)	(Periasamy et al., 2013)	
*Lipid mixtures*			
Soybean total extract, containing 20% lecithin	Human stearoyl-CoA desaturase complex	(Goren and Fox, 2008) (Goren et al., 2009)	Functional and structural analysis by NMR
	TbSLS4 (*Trypanosoma brucei* sphingolipid synthase 4)	(Sevova et al., 2010)	Enzymatic specificity analysis
	PilD (*Pseudomonas eruginosa* prepilin peptidase)	(Aly et al., 2012)	Enzymatic activity
	*Shaker* potassium channels	(Jarecki et al., 2013)	Oocyte injection
	AtRGS1 (*Arabidopsis thaliana* regulator of G protein Signaling 1)	(Li et al., 2016)	Functional and biochemical analysis
DMPC/cholesterol (70/30, mol/mol)	Cytochrome b_5_	(Nomura et al., 2008)	Transport activity
DOPG/POPE (2/3, w/w)	CrdS (*Agrobacterium* curdlan synthase)	(Periasamy et al., 2013)	
POPC/CL/POPS/POPA (54/24/16/4/2, mol/mol)	AAC (ADP/ATP carrier)	(Long et al., 2012)	Transport activity
*E. coli* lipids	CrdS (*Agrobacterium* curdlan synthase)	(Periasamy et al., 2013)	
*Lipid/detergent mixtures*			
Asolectin liposomes Brij-35	AtPPT1 (*Arabidopsis thaliana* phosphoenolpyruvate/phosphate translocator 1)	(Nozawa et al., 2007)	
*Other mixtures*			
Asolectin/20% glycerol (glycerosome)	HRH1 (human histamine H1 receptor, GPCR)	(Suzuki, 2018)	New drug delivery system

*Nanodisc*			
MSP1D1 Cardiolipin	AtRGS1 (*Arabidopsis thaliana* regulator of G protein Signaling 1) Arabidopsis thaliana receptor-like kinase (RLK) FERONIA	(Li et al., 2016) (Fogeron et al., 2015a; Fogeron et al., 2015b; Li et al., 2016; Fogeron et al., 2017a; Minkoff et al., 2017)	Functional and biochemical analysis Functional and structural characterization
MSP1E3D1 POPC/POPE/tocl (40/40/20, mol/mol)	Tim23 (subunit of the TIM23 protein transport complex)	(Malhotra and Alder, 2017)	Functional and structural characterization

Liposomes. DOPG (1,2-dioleoyl-sn-glycero-3-phospho-(1′-rac-glycerol)); DOTAP, 1,2-dioleoyl-3-trimethylammonium-propane; DMPC, 1,2-dimyristoyl-sn-glycero-3-phosphocholine; DOPC, 1,2-di-oleoyl-sn-glycero-3-phosphocholine; EYPC, egg yolk phosphatidylcholine; POPC, 1-palmitoyl-2-oleoyl-sn-glycero-3-phosphocholine; POPE, 1-palmitoyl-2-oleoyl-sn-glycero-3-phosphoethanolamine; CL, 19,39-bis[1,2-dioleoyl-sn-glycero-3-phospho]-sn-glycerol; POPS, 1-palmitoyl-2-oleoyl-sn-glycero-3-phosphoserine; POPA, 1-palmitoyl-2-oleoyl-sn-glycero-3-phosphate.

Nanodiscs. MSP1D1, membrane scaffold protein 1D1; MSP1E3D1, membrane scaffold protein 1E3D1; TOCL, 1′,3′-bis[1,2-dioleoyl-sn-glycerol-3-phospho-]-sn-glycerol.

Another possibility is the addition of microsomes, which are membranous vesicles obtained from the ER often from dog pancreas (Jackson and Blobel, 1977), oocytes (Kobilka, 1990; Lyford and Rosenberg, 1999), or oviduct cells (Rosenberg and East, 1992). Canine pancreatic microsomal membranes are commercially available (Promega, United States) and allow for signal peptide cleavage, membrane insertion, translocation, and core glycosylation according to the maker. In the presence of microsomes, membrane proteins having a signal peptide are translocated through the translocon of the ER membrane and then can be glycosylated within the lumen of the membranes (Dobberstein and Blobel, 1977; Katz et al., 1977; Lingappa et al., 1978). Since the protein synthesis machinery is present only outside the vesicles, a prevalent inside-out orientation of membrane proteins can be expected (Schwarz et al., 2008). There are, however, few reports on this approach using the WGS (Dobberstein and Blobel, 1977; Jackson and Blobel, 1977; Katz et al., 1977; Lingappa et al., 1978), mainly because of low expression yields making this approach only suitable for functional protein analyses.

Alternatively, cotranslation insertion in a synthetic membrane of block copolymer vesicles has been described for the CXCR4 GPCR (De Hoog et al., 2014). Other alternatives to liposomes and biological membrane vesicles are bicelles and nanodiscs (Ritchie et al., 2009; Bayburt and Sligar, 2010; Dürr et al., 2012; Lyukmanova et al., 2012a; Sachse et al., 2014). The diameter of nanodiscs ranges from 10 to 20 nm, depending on the length and type of the membrane scaffold protein (Sachse et al., 2014). During synthesis, membrane proteins are incorporated into nanodiscs in a passive manner and can later on be extracted from them in a native functional form (Ranaghan et al., 2011). A major advantage is that nanodiscs keep membrane proteins soluble in a detergent-free environment, possibly yielding monodisperse and homogenous samples (Borch and Hamann, 2009; Henrich et al., 2015; Danmaliki and Hwang, 2020). A tag fused to the membrane scaffold protein allows moreover for a simple purification procedure (Bayburt and Sligar, 2010). Recent examples of membrane proteins produced in the presence of nanodiscs using the WGS are summarized in Table 5. This includes, for example, the synthesis of the G protein Signaling 1 (AtRGS1) protein from *Arabidopsis thaliana* (Li et al., 2016). A major drawback of nanodiscs in solid-state NMR is that they might result in low signal-to-noise ratios due to high lipid-to-protein ratios (Jirasko et al., 2020). For solution NMR, optimized nanodiscs have been developed with smaller diameters (Hagn et al., 2013). Nanodiscs can be unstable, and therefore polymer-enhanced versions have been described to extend the use of this promising platform for studies on membrane proteins (Chen et al., 2020).

To summarize, there are different alternatives to produce membrane proteins in a native form. The most suitable one depends mainly on the nature of the protein and the final application. For our purposes in NMR sample preparation, the expression directly in a detergent-solubilized form, followed by affinity purification and lipid reconstitution, has given the most convincing results, since it also allowed the selection of a lipid-to-protein ratio which minimizes the amount of lipids in NMR rotors (Lacabanne et al., 2019; Jirasko et al., 2020). In one special case, membrane envelopes of the duck hepatitis B virus were autoassembled when using the WGS in the presence of mild detergents, likely using lipids present in the wheat germ extract (David et al., 2018), which made reconstitution dispensable.

Chaperones

Molecular chaperones are important protein factors often needed for correct conformational folding of proteins. A recombinant *E. coli* CFPS system, the PURE system, was used to systematically test the impact of chaperones on the solubility of ∼800 proteins (Niwa et al., 2012) without interference of other proteins from a cell extract, showing their importance to improve protein quality. The eukaryotic translation machinery is thought to have been optimized through evolution to support cotranslational protein folding (Endo and Sawasaki, 2006). Newly synthesized proteins in the WGS can indeed be stabilized by eukaryotic chaperones promoting folding. This is best documented for the formation of disulfide bonds. Since translation reaction buffers commonly contain the reducing agent dithiothreitol (DTT), the production of disulfide bond-containing proteins is a delicate issue. However, lowering DTT concentration commonly leads to decreased expression yields, although disulfide bond formation was demonstrated in a DTT-deficient reaction in the presence of a protein disulfide isomerase (PDI) (Kawasaki et al., 2003). The addition of chaperones that catalyze disulfide bond formation to the translation reaction therefore might be necessary for correct folding (Saaranen and Ruddock, 2019). As an example, the cooperative role of quiescin sulfhydryl oxidase (QSOX) and PDI was investigated for the generation of native pairings in unfolded reduced proteins (Rancy and Thorpe, 2008; Gad et al., 2013). Interestingly, glutathione-based redox buffers are not needed for efficient folding using a combination of QSOX and PDI (Rancy and Thorpe, 2008). If used, the ratio of reduced and oxidized glutathione might have to be optimized depending on the protein of interest. The use of a combination of a PDI and Ero1α system (Inaba et al., 2010; Shergalis et al., 2020) was established by CellFree Sciences for the synthesis of an anti-AGIA-IgG antibody Fab fragment exhibiting antigen-binding capacity after protein synthesis (https://www.cfsciences.com/eg/resources/application-note/519-note-9). In addition to using exogenous chaperones, pretreatment of *E. coli* extracts with the alkylating agent iodoacetamide (IAA) which covalently blocks the free sulfhydryl groups of enzymes has been reported to support oxidative folding (Kim and Swartz, 2003; Yin and Swartz, 2004). This approach could be considered for the WGS as well to prevent aggregation and to improve both solubility and activity, yielding more of the disulfide bond-containing proteins. Fine-tuning of redox conditions for disulfide bond formation has been described for human and mouse prion-like Doppel proteins and mouse interleukin-22 (Michel and Wuthrich, 2012) and has also proven to be efficient for disulfide bond formation in virus-like particles (Bundy and Swartz, 2011), all these proteins being produced using *E. coli* lysates. This approach was further shown to support the formation of intramolecular disulfide bonds during the synthesis of antibody fragments using an insect cell lysate (Stech et al., 2014). Although there are few data available about this approach for the WGS (Rancy and Thorpe, 2008; Gad et al., 2013), it could definitely be of interest when disulfide bond formation is desired. Since disulfide bonds are commonly found under oxidizing conditions, further considerations should be given to the storage and processing of proteins carrying this modification. Several methods have been published for the analysis of disulfide bonds including Ellman’s reagent [5,5′-dithiobis-(2-nitrobenzoic acid) or DTNB] used to quantify the concentration of thiol groups (Ellman, 1959; Winther and Thorpe, 2014), mass spectrometry (Lakbub et al., 2018), NMR (Denisov et al., 2019; Wiedemann et al., 2020), or simpler methods using PEG-maleimide to modify free thiol groups. Proteins may also be analyzed under nonreducing and reducing conditions by SDS-PAGE (Braakman et al., 2017). However, posttranslational formation of disulfide bonds in CFPS experiments or later during processing of proteins has its own challenges as not every cysteine may form correct bonds. As a first indication for correct folding, we suggest looking for an increase in protein solubility and, where possible, to confirm protein activity in a functional test or take an NMR spectrum.

The chaperone function of Ric-8 proteins was shown to be required for proper folding of heterodimeric G proteins (Chan et al., 2013). More recently, it was also shown that coexpression of J-domain containing chaperone proteins with potassium channels is essential for their folding, stabilization, and tetrameric assembly (Li et al., 2017). Another example of the correlation between cofactor binding and protein folding was demonstrated for the Flavin Mono Nucleotide- (FMN-) binding protein (Abe et al., 2004). The WGS allows for coexpression of two or more proteins in the same translation reaction, where the expression of binding partners may assist proper expression. Direct preparation of protein complexes in cotranslation experiments will open new ways to make use of chaperones to assist the production of functional proteins.

## Large-Scale Protein Production

Cell-free protein expression systems can use different reaction formats, and the choice of the best suited reaction format depends on the application. This can include functional and structural investigations for research or clinical purposes, small-scale assays, high-throughput screening or large-scale production including industrial use for diagnostic or therapeutic applications. Commonly, protein quantities for structural and functional studies or antigen production are in the range of milligram amounts, while much larger quantities could be desired for industrial applications. All those needs have been achieved by different CFPS. Therefore, the main parameters to be considered include the size and nature of the protein of interest, ease of implementation, productivity, reaction time, ability to scale, and the cost of the platform. To meet those needs, WG-CFPS reactions can be performed in either one-compartment or two-compartment reactions, and final yields between 1 and 20 mg of GFP per mL wheat germ extract can be achieved using high-quality extracts (Harbers, 2014).

The batch mode (Kawasaki et al., 2003) is a one-compartment reaction in which all reagents are mixed in a single container and is thereby the least complicated. The system works, however, only for a few hours, mainly due to the accumulation of inhibitory byproducts in the single reaction compartment (Schwarz et al., 2008), and the amount of the synthesized protein is usually not sufficient for structural investigations (Sawasaki et al., 2002a). The batch mode is ideal for small-scale high-throughput expression screening experiments (Sawasaki et al., 2002a; Schwarz et al., 2008). One alternative to the regular batch reaction format is the so-called repeat-batch or discontinuous batch mode (Harbers, 2014). After incubation, the batch reaction is concentrated by a centrifugation step; then, fresh reaction buffer is added to provide new substrates. Multiple concentration cycles can thus be performed, leading to higher protein yields than the batch mode (Harbers, 2014). An automated discontinuous batch system was described for the production of soluble *Galdieria sulphuraria* protein DCN1, leading to a yield higher than 2 mg/mL in the reaction mixture allowing for its structure determination by X-ray crystallography from a 10 mL reaction (Beebe et al., 2011). This approach has also been applied to the production of membrane proteins in the presence of detergents or lipids (Beebe et al., 2011).

**FIGURE 5:**
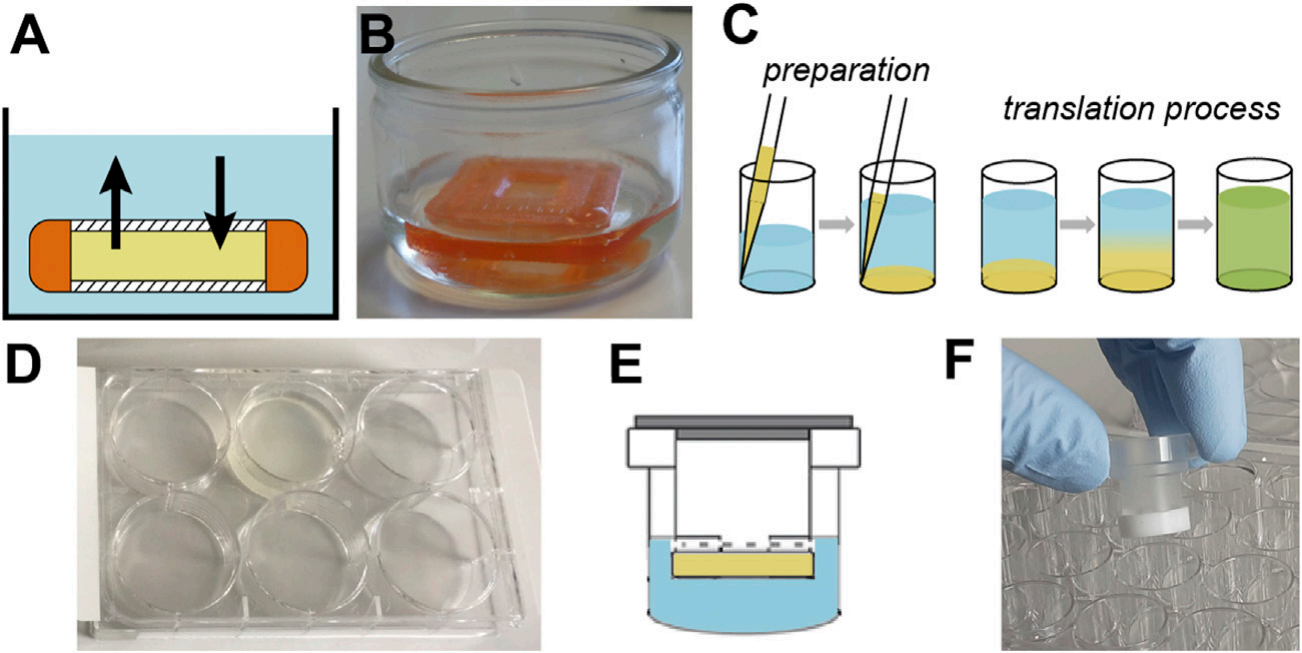
Different reaction formats for protein expression using the WGS. **(A)** Schematic representation and **(B)** picture of a 500 μL dialysis cassette for medium scale CECF production. **(C)** Schematic representation of the bilayer method which is performed either in a 96-well plate for small-scale expression test or in a 6-well plate for larger-scale production **(D)**, adapted from Fogeron et al. (2015a), Fogeron et al. (2015b); Fogeron et al. (2017b). **(E)** Schematic representation of the dialysis method and **(F)** picture of a CECF mini-reactor manufactured at ETH Zurich by Andreas Hunkeler in Beat H. Meier’s laboratory, according to Schneider et al. (2009). In this reaction format, a 24-well plate is used. For all panels, the translation mix is represented in yellow while the feeding buffer is represented in blue.

The continuous-exchange cell-free (CECF) system is a two-compartment setup in which the cell-free protein expression reaction is separated from the feeding buffer by a semipermeable membrane (Katzen et al., 2005). The cell-free expression reaction takes place in the dialysis device, and the dialysis buffer containing fresh substrates can diffuse in, while byproducts passively diffuse out (Figures 5A,B). Mini- and maxi-CECF reactors, as well as further CECF reactor designs, have been described in detail (Schneider et al., 2009). Interestingly, a microfluidic platform was also described for CECF, allowing for reduced reaction volumes and simultaneous expression of up to 96 proteins (Jackson et al., 2014). Making proteins for NMR use, we standardly use 500 μL and 3 mL commercial dialysis cassettes and the CECF system (David et al., 2018; Wang et al., 2019) (Figures 5A,B). When larger protein amounts are needed, larger dialysis cassettes can be used, or more generally, CECF reactions can be run in parallel as described in (Aoki et al., 2009).

The continuous-flow cell-free (CFCF) system is another two-compartment setup like CECF. First described by Spirin (Spirin et al., 1988; Spirin, 2004), the CFCF system provides, through the use of a pump, the automated and continuous supply of substrates into the reaction chamber and the removal of byproducts which are pushed out through an ultrafiltration membrane, simultaneously retaining the protein of interest (Endo et al., 1992). The translation reaction can thus proceed for more than two days, which is more than ten times longer than the batch mode, and can yield up to several milligrams of protein (Spirin et al., 1988; Morita et al., 2003). This approach has been reported to be not suitable for high molecular weight proteins over 300 kDa (Spirin, 2004). Continuous reaction formats such as CECF and CFCF are nonetheless attractive for industrial protein production, and automated systems have been optimized in this direction (Vinarov et al., 2006a; Aoki et al., 2009; Revathi et al., 2010).

The bilayer method is a simplified and less expensive version of CECF and CFCF (Sawasaki et al., 2002a). In contrast to the CECF and CFCF systems, the two compartments are not separated by a semipermeable membrane, and the total reaction has thus to be harvested for further analysis (Figure 5C). This method allows for the synthesis of protein amounts compatible with functional and structural analyses. The substrate buffer is overlaid onto the translation mixture, forming two separate layers through their different density, thus allowing for a diffusion-controlled translation process (Sawasaki et al., 2002a; Takai et al., 2010). The bilayer method can be fully automated for large-scale and efficient screening (Endo and Sawasaki, 2004; Vinarov et al., 2006b). Moreover, its flexible format permits screening different additives for the translation reaction, such as detergents or lipids for the expression of membrane proteins in a soluble form (Harbers, 2014), and can be readily scaled up from 96-well plates to 6-well plates (Figure 5D). Over 13,000 human cDNA clones have already been tested for protein expression using this method (Goshima et al., 2008). Bilayer expression is easier to handle and less expensive than the CECF and CFCF modes but much more efficient than batch reactions. While yields are about three times higher in the dialysis mode, the cost is proportionally higher in the case of protein labeling because of the larger buffer volume. In addition, solubility could be an issue in the dialysis mode since proteins are more concentrated.

For much higher throughput, protein synthesis using microfluidics approaches was also described for the WGS and has been used for the kinetic analysis of transcription factor-DNA interactions (Geertz et al., 2012) and to perform 96 dialysis reactions in parallel. The microfluidics approach can improve protein expression, offering much higher yields as compared to batch reactions (Jackson et al., 2014). These methods have the potential to become more important in the context of biomedical and diagnostic approaches as well as applications in systems biology looking at many proteins at the same time (Ayoubi-Joshaghani et al., 2020).

In the context of NMR sample preparation, we mainly use the bilayer method for small-scale expression tests and screening of additives in 96-well plates (Fogeron et al., 2015b). We have also implemented the mini-CECF reactor (Schneider et al., 2009) in our laboratory for samples that need to be more concentrated for analysis (Figures 5E,F, unpublished). When sample concentration is not an issue, the bilayer method is definitely the method of choice when working at a small scale and to reduce the volumes needed for translation reactions and feeding buffer or while adding stable isotope-labeled amino acids. For larger-scale production of labeled NMR samples, we usually perform the translation reaction either using the bilayer method in 6-well plates followed by affinity purification (Fogeron et al., 2015b) or in the CECF mode using dialysis cassettes followed by isolation of the protein on a density gradient in the case of proteoliposomes, capsids, or viral envelope assemblies (David et al., 2018; Wang et al., 2019). In our hands, proteins in this setup typically yield between 0.2 and 1 mg protein per mL wheat germ extract used. The cost for this screening is around 300 € per reaction using 1 mL wheat germ extract (including purification), where the triply labeled amino acids only represent about 120 €. For 1 mg of protein, sufficient for solution and solid-state NMR experiments (0.7 mm rotor), this results in a cost of 600–1,200 € for a ^2^H/^13^C/^15^N labeled sample. When using commercial extracts, the cost of the extract must be added to this. The production of home-made wheat germ extracts can be done at negligible cost when only reagents are considered. Our lab routinely produces around 100 mL wheat germ extract per year, with eye sorting of the wheat germs done on a single day every two months by the entire group (<10 people). The extract is then prepared in two days by one person (Fogeron et al., 2015a). In bacterial expression, a triply ^2^H/^13^C/^15^N labeled protein preparation costs around 1700 €/L culture; the sample cost then depends on the yields which can be achieved but for complex systems are often not above 1–5 mg/L. Costs for triply labeled samples are thus rather similar, until proteins express with high yield in bacteria. ^13^C/^15^N labeling in WG-CFPS is not much less costly than triple labeling and reduces the cost of amino acids by only a factor of two, whereas in bacterial expression, the factor is nearly ten. Therefore, it makes most sense to use CFPS for complex proteins where bacterial expression fails and in cases where deuteration is of high importance.

## Application to Structural Biology

**FIGURE 6:**
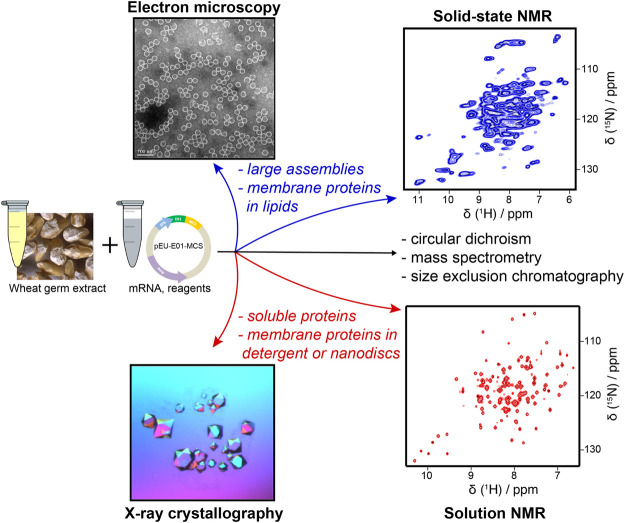
Structural characterization of proteins produced from cell-free protein expression.

While CFPS is important for a variety of applications, we will give some examples from structural biology (Figure 6), where most experiments are done on recombinant proteins. While in many cases *E. coli* expression gives satisfactory results with respect to yield, it does not produce well-folded proteins in all cases. From experience, expression in cells corresponding to the origin of the protein (e.g., often mammalian cells) would be the most adapted approach with respect to correct folding; though, yield is often prohibitively low. CFPS, notably using eukaryotic systems, is a good compromise for mammalian proteins, providing sufficient yield and in many cases correct folding. The eukaryotic ribosomes are also ensuring a slower synthesis as compared to bacterial systems, which in turn promotes cotranslational folding. Furthermore, CFPS-generated proteins are easy to purify, which is important for crystallographic studies. The WGS can easily provide around 50 μg of protein needed for cryo-EM and even more than the minimum of around 1 mg needed for X-ray crystallography and NMR. The following section illustrates some examples of how WGS was successfully applied in the past years for structural biology approaches, including solution and solid-state NMR, cryo-EM, and X-ray crystallography.

### NMR

WGS is particularly attractive for its ability to produce complex eukaryotic (membrane) proteins for NMR and importantly combines this with the major advantage other CFPSs have, that is, the efficient and specific isotopic labeling required for NMR protein studies. Indeed, only the cell-free synthesized protein is isotopically labeled during expression (Morita et al., 2003); therefore, even if some remaining contaminants are present in the sample, they will be invisible in the NMR spectra. Amino acid selective labeling, easily implemented in CFPS by simply adding the desired amino acids into the reaction mixture, can also be used in WGS (Morita et al., 2004; Kohno and Endo, 2007; Tonelli et al., 2011; Fogeron et al., 2015a; Jirasko et al., 2020). Selective labeling results in significantly reduced NMR spectral complexity and enables application to higher molecular weight systems (>50 kDa) (Tugarinov et al., 2006). In the case of WGS, ^15^N-selective labeling was shown to be efficient for most amino acids, except for Ala, Glu, and Asp (Morita et al., 2004). For these residues, the addition of inhibitors for transaminases and glutamine synthase during protein synthesis is required to avoid scrambling (transfer of isotope labels between amino acids) (Morita et al., 2004). This method was first demonstrated on the RNA-binding protein RbpA1 and yeast ubiquitin (Morita et al., 2004) and then successfully applied to produce specific labeling schemes in β2-microglobulin (Kameda et al., 2009), a structural component of amyloid fibrils. The specific labeling scheme was crucial in the structural characterization of the refolding intermediate of β2-microglobulin and enabled to reveal the regions important for amyloidogenicity (Kameda et al., 2009). Another example is the ^15^N-Val selectively labeled yeast ubiquitin, where the four valine residues could be observed in the ^1^H–^15^N HSQC spectrum (Kohno, 2010).

For NMR studies, it is sometimes necessary to deuterate the protein, except for the amide protons, to improve the spectral linewidth. In the WGS, deuterated amino acids can be used, while the expression is done in H_2_O to obtain labeling. Hence, there is no need for a posteriori proton back exchange as in cell-based expression, since amide protonation is achieved directly during synthesis, avoiding a denaturation and refolding step, which can compromise the native fold of proteins. The usefulness of this approach was reported in the case of HBV capsids, where 20% of amide protons from the hydrophobic core are missing when bacterial expression is used (Lecoq et al., 2019), while they are present in samples prepared by WG-CFPS (Lecoq et al., 2019). It has been shown that metabolic scrambling cannot be avoided, and proton back exchange can occur on CH groups of Gly, Ala, Asp, Glu, Gln, and Lys (Tonelli et al., 2011). This problem can be alleviated by using similar transaminase inhibitors (Morita et al., 2004; Tonelli et al., 2011). The WGS and NMR were also applied to NS5A from the hepatitis C virus (HCV), where it revealed phosphorylation sites on the protein (Badillo et al., 2017).

**FIGURE 7:**
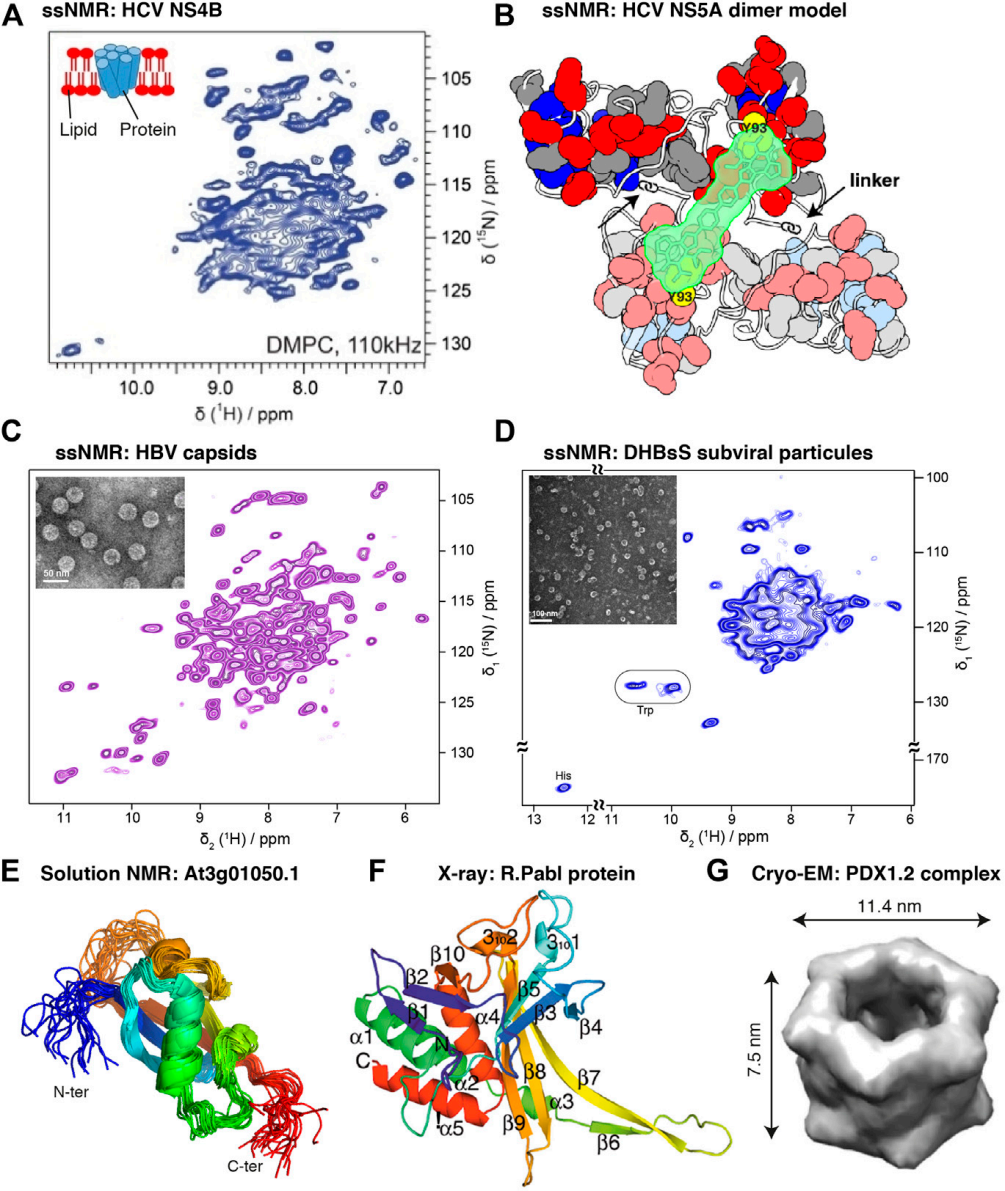
Examples of structural studies on proteins expressed in WGS using NMR, X-ray crystallography, and cryo-EM. **(A)** Solid-state NMR spectrum of the HCV membrane protein NS4B reconstituted into DMPC lipids (Jirasko et al., 2020). **(B)** Dimer orientation in lipids of the HCV helix anchor and domain 1 (AHD1) of the NS5A protein as determined by solid-state NMR (Jirasko et al., 2020). **(C)** Solid-state NMR spectra of the hepatitis B virus capsid (Wang et al., 2019) and of **(D)** the subviral particles made of duck HBV small envelope protein (DHBs S) (David et al., 2018). The three spectra have been recorded at 110 kHz MAS on an 850 MHz spectrometer. Both HBV capsids and subviral particles were autoassembled during cell-free synthesis; their negative-staining electron microscopy images are shown inside the corresponding spectrum. **(E)** 20 conformers obtained by solution NMR of At3g01050.1 protein (Vinarov et al., 2004) (PDB 1se9, figure prepared using PyMoL (https://pymol.org/2/). **(F)** Structure of restriction endonuclease PabI obtained by X-ray crystallography (Miyazono et al., 2007; Watanabe et al., 2010) (PDB 2dvy). **(G)** 3D cryo-EM reconstruction of PDX1.2 complex at 15 Å resolution (Novikova et al., 2018). Figures were adapted with permission from Jirasko et al. (2020) for panel A, from Wang et al. (2019) for panel C, and from David et al. (2018) for panel D and reprinted with permission from Jirasko et al. (2020) for panel D, from Miyazono et al. (2007), Watanabe et al. (2010) for panel F, and from Novikova et al. (2018) for panel **G**.

With advances in MAS solid-state NMR, membrane proteins can be studied in lipids (reviewed in (Ladizhansky, 2017)). Recently, MAS frequencies exceeding 100 kHz have allowed structural investigation of submilligram amounts of protein (Agarwal et al., 2014; Andreas et al., 2016), as typically can be produced using WGS. This has enabled studies of HCV NS4B (Figure 7A) (Fogeron et al., 2016; Jirasko et al., 2020) and NS5A. NMR could show that the NS5A dimer in lipids presented a different orientation than in crystals and a model could be forwarded which proposes a binding mode for the directly acting antiviral Daclatasvir (Jirasko et al., 2020) (Figure 7B). It was also shown that high-quality NMR spectra can be obtained on HBV capsids (Figure 7C) and envelope proteins (the latter from the duck virus variant) proteins (Figure 7D) (David et al., 2018; Wang et al., 2019) which are spontaneously self-assembled in the WGS (Lingappa et al., 2005; David et al., 2018; Wang et al., 2019). For the HBV capsid, the combination of WGS and solid-state NMR allowed studying the effect of capsid assembly modulators at the exit from the ribosome (Wang et al., 2019).

Solution NMR applications of the WGS were advanced in the field of structural genomics by Markley and coworkers, who used it to screen 238 eukaryotic hypothetical proteins from *A. thaliana* and human genomes (Vinarov et al., 2004). Nearly half of these proteins were found to be soluble, and 40% yielded ^1^H–^15^N HSQC spectra indicative of folded proteins. Several solution NMR structures were solved of WG-CF-synthesized proteins, including the *A. thaliana* protein At3g01050.1 (Figure 7E) (Vinarov et al., 2004). A detailed comparison of *E. coli* expression with the WG-CFPS revealed that solubility and folding reached a higher success rate in the WGS (Tyler et al., 2005). The implementation of a high-throughput cell-free translation platform at the Center for Eukaryotic Structural Genomics enabled the use of WGS for fast screening and solution NMR structural determination (Makino et al., 2010; Makino et al., 2014; Vinarov and Markley, 2014).

Another recent work reports WG-CFPS of virtually all SARS-CoV-2 accessory proteins and M and E structural proteins, which showed that most of them can be produced and purified in soluble form and in milligram amounts (Altincekic et al., 2021)^1^.

### X-Ray Crystallography and Cryo-EM

The first X-ray structure of a protein expressed in the WGS was solved in 2007 (Miyazono et al., 2007; Watanabe et al., 2010), on the cytotoxic R.PabI (Figure 7F), one of the 4 bp cutter restriction enzymes, which are highly toxic to the cells. Recent literature shows further examples (Novikova et al., 2018), including the hexameric assembly of the dioxide-concentrating mechanism protein (CCMK) (Novikova et al., 2018). Also, diffracting crystals of the glutamine synthetase from *O. tauri* complex were obtained from a crystallization screen using WGS (Novikova et al., 2018). Further negative-stain EM data provide indications that autoassemblies of larger superstructures are commonly observed when working with WGS, without showing aggregation nor disordered complexes, suggesting highly homogeneous samples compatible with high-resolution studies (Novikova et al., 2018). Furthermore, the EM envelope of the pyridoxal 5′-phosphate synthase-like subunit (PDX1.2) from *A. thaliana* (Figure 7G) was solved (Novikova et al., 2018). As WGS application for cryo-EM is now just emerging, we expect a much wider use of WGS for preparation of samples for cryo-EM studies.

## Discussion

Today, CFPS is a mature technology that can serve most needs in protein expression. In recent years, the technology has made interesting contributions to the new fields of synthetic biology and the development of minimal cells. In contrast to *in vivo* expression systems, CFPS reactions enable manipulation of the reaction conditions in accordance with the requirements of a given protein. They can also be implemented in fully automated protein production to obtain larger yields or throughput and are thus a suitable approach to rapidly screen many mutations for changes to protein function and later production on a larger scale to obtain structural data. A former disadvantage of WG-CFPS used for structural studies was the lower yield when compared to bacterial cell-free or cell-based expression. However, structural studies currently need less protein, which makes these lower yields less of a barrier. Therefore, WGS today is fully compatible with high-resolution structural biology methods such as cryo-EM, X-ray crystallography, solution NMR, and solid-state NMR. The unique possibilities of this system for studies on cytotoxic proteins (Miyazono et al., 2007), complex membrane proteins (Fogeron et al., 2016; Badillo et al., 2017; David et al., 2018), or molecular assemblies in a native-close state shall thus create opportunities for structural approaches to study complex and difficult proteins. The examples given in this review demonstrate that CFPS production can be a powerful alternative to cell-based methods and thus could enable entirely new applications.

With recent developments in bioinformatics and methods of applied synthetic biology, we foresee rapid progress for new approaches to optimize protein expression using CFPS systems. These approaches will take advantage of computational protein design (Gustafsson et al., 2012), rapid template generation by gene synthesis, and working with linear DNA templates. Such linear templates can be used in fully automated and highly parallel testing of different template/protein designs and translation reaction conditions using robotic or microfluidic devices to perform effective expression tests yielding optimized proteins and conditions for their functional synthesis in a truly short timeframe (Ayoubi-Joshaghani et al., 2020; Borkowski et al., 2020). Conditions can subsequently be scaled to produce the protein for further use. This progress will largely be driven by the unique features of CFPS systems and the freedom they offer to adopt the system as this is already done for rational biodesigns (Laohakunakorn, 2020). Another important aspect for future developments will be the production of proteins having posttranslational modifications, where approaches have already been discussed for tyrosine and serine phosphorylation, lysine acetylation, and lysine methylation (Venkat et al., 2019). Similarly, we expect more progress on protein glycosylation and engineering glycosylation reactions in CFPS (Hershewe et al., 2020; Kightlinger et al., 2020), a key aspect for making biologicals for treatment. With this outlook, we expect remarkably interesting development`s for new methods and applications that will involve a CFPS step as an essential part of the assay system. We hope that the WGS will contribute to these developments as it proved to be one of the most effective eukaryotic CFPS systems.

## Footnotes

1 Altincekic, N., Korn, S.M., Qureshi, N.S. Dujardin, M., Ninot-Pedrosa, M., Abele, R., et al. Front. Mol. Biosc., in production.

## References

[B1] Abe M. Hori H. Nakanishi T. Arisaka F. Ogasawara T. Sawasaki T. (2004). Application of cell-free translation systems to studies of cofactor binding proteins. Nucleic Acids Symp. Ser. 13, 143–144. 10.1093/nass/48.1.143 17150519

[B2] Aceti D. J. Bingman C. A. Wrobel R. L. Frederick R. O. Makino S. Nichols K. W. (2015). Expression platforms for producing eukaryotic proteins: a comparison of *E. coli* cell-based and wheat germ cell-free synthesis, affinity and solubility tags, and cloning strategies. J. Struct. Funct. Genomics 16, 67–80. 10.1007/s10969-015-9198-1 25854603 PMC4430420

[B3] Agarwal V. Penzel S. Szekely K. Cadalbert R. Testori E. Oss A. (2014). De novo 3D structure determination from sub-milligram protein samples by solid-state 100 kHz MAS NMR spectroscopy. Angew. Chem. Int. Ed. Engl. 53, 12253–12256. 10.1002/anie.201405730 25225004

[B4] Akbarzadeh A. Rezaei-Sadabady R. Davaran S. Joo S. W. Zarghami N. Hanifehpour Y. (2013). Liposome: classification, preparation, and applications. Nanoscale Res. Lett. 8, 102. 10.1186/1556-276X-8-102 23432972 PMC3599573

[B5] Althoff T. Davies K. M. Schulze S. Joos F. Kühlbrandt W. (2012). GRecon: a method for the lipid reconstitution of membrane proteins. Angew. Chem. Int. Ed. Engl. 51, 8343–8347. 10.1002/anie.201202094 22821803 PMC3494379

[B6] Aly K. A. Beebe E. T. Chan C. H. Goren M. A. Sepúlveda C. Makino S. (2012). Cell-free production of integral membrane aspartic acid proteases reveals zinc-dependent methyltransferase activity of the *Pseudomonas aeruginosa* prepilin peptidase PilD. MicrobiologyOpen 2, 94–104. 10.1002/mbo3.51 23255525 PMC3584216

[B7] Anastasina M. Terenin I. Butcher S. J. Kainov D. E. (2014). A technique to increase protein yield in a rabbit reticulocyte lysate translation system. Biotechniques 56, 36–39. 10.2144/000114125 24447137

[B8] Andreas L. B. Jaudzems K. Stanek J. Lalli D. Bertarello A. Le Marchand T. (2016). Structure of fully protonated proteins by proton-detected magic-angle spinning NMR. Proc. Natl. Acad. Sci. U.S.A. 113, 9187–9192. 10.1073/pnas.1602248113 27489348 PMC4995937

[B9] Aoki M. Matsuda T. Tomo Y. Miyata Y. Inoue M. Kigawa T. (2009). Automated system for high-throughput protein production using the dialysis cell-free method. Protein Expr. Purif. 68, 128–136. 10.1016/j.pep.2009.07.017 19664715

[B10] Arimitsu E. Ogasawara T. Takeda H. Sawasaki T. Ikeda Y. Hiasa Y. (2014). The ligand binding ability of dopamine D1 receptors synthesized using a wheat germ cell-free protein synthesis system with liposomes. Eur. J. Pharmacol. 745, 117–122. 10.1016/j.ejphar.2014.10.011 25446930

[B11] Arinaminpathy Y. Khurana E. Engelman D. M. Gerstein M. B. (2009). Computational analysis of membrane proteins: the largest class of drug targets. Drug Discov. Today 14, 1130–1135. 10.1016/j.drudis.2009.08.006 19733256 PMC2796609

[B12] Arranz-Gibert P. Vanderschuren K. Isaacs F. J. (2018). Next-generation genetic code expansion. Curr. Opin. Chem. Biol. 46, 203–211. 10.1016/j.cbpa.2018.07.020 30072242 PMC6748649

[B13] Arumugam T. U. Ito D. Takashima E. Tachibana M. Ishino T. Torii M. (2014). Application of wheat germ cell-free protein expression system for novel malaria vaccine candidate discovery. Expert Rev. Vaccin. 13, 75–85. 10.1586/14760584.2014.861747 24308585

[B14] Athey J. Alexaki A. Osipova E. Rostovtsev A. Santana-Quintero L. V. Katneni U. (2017). A new and updated resource for codon usage tables. BMC Bioinform. 18, 391. 10.1186/s12859-017-1793-7 PMC5581930 28865429

[B15] Ayoubi-Joshaghani M. H. Dianat-Moghadam H. Seidi K. Jahanban-Esfahalan A. Zare P. Jahanban-Esfahlan R. (2020). Cell-free protein synthesis: the transition from batch reactions to minimal cells and microfluidic devices. Biotechnol. Bioeng. 117, 1204–1229. 10.1002/bit.27248 31840797

[B16] Badillo A. Receveur-Brechot V. Sarrazin S. Cantrelle F. X. Delolme F. Fogeron M. L. (2017). Overall structural model of NS5A protein from hepatitis C virus and modulation by mutations confering resistance of virus replication to cyclosporin A. Biochemistry 56, 3029–3048. 10.1021/acs.biochem.7b00212 28535337

[B17] Banerjee R. K. Datta A. G. (1983). Proteoliposome as the model for the study of membrane-bound enzymes and transport proteins. Mol. Cel. Biochem. 50, 3–15. 10.1007/BF00225276 6221188

[B18] Bardóczy V. Géczi V. Sawasaki T. Endo Y. Mészáros T. (2008). A set of ligation-independent *in vitro* translation vectors for eukaryotic protein production. BMC Biotechnol. 8, 32. 10.1186/1472-6750-8-32 18371187 PMC2311287

[B19] Bayburt T. H. Sligar S. G. (2010). Membrane protein assembly into Nanodiscs. FEBS Lett. 584, 1721–1727. 10.1016/j.febslet.2009.10.024 19836392 PMC4758813

[B20] Beebe E. T. Makino S. Nozawa A. Matsubara Y. Frederick R. O. Primm J. G. (2011). Robotic large-scale application of wheat cell-free translation to structural studies including membrane proteins. N. Biotechnol. 28, 239–249. 10.1016/j.nbt.2010.07.003 20637905 PMC3035758

[B21] Berg M. D. Brandl C. J. (2020). Transfer RNAs: diversity in form and function. RNA Biol. 12, 1–24. 10.1080/15476286.2020.1809197 PMC7954030 32900285

[B22] Berrier C. Park K. H. Abes S. Bibonne A. Betton J. M. Ghazi A. (2004). Cell-free synthesis of a functional ion channel in the absence of a membrane and in the presence of detergent. Biochemistry 43, 12585–12591. 10.1021/bi049049y 15449948

[B23] Blesneac I. Ravaud S. Juillan-Binard C. Barret L. A. Zoonens M. Polidori A. (2012). Production of UCP1 a membrane protein from the inner mitochondrial membrane using the cell free expression system in the presence of a fluorinated surfactant. Biochim. Biophys. Acta 1818, 798–805. 10.1016/j.bbamem.2011.12.016 22226924

[B24] Böckmann A. Ernst M. Meier B. H. (2015). Spinning proteins, the faster, the better?. J. Magn. Reson. 253, 71–79. 10.1016/j.jmr.2015.01.012 25797006

[B25] Borch J. Hamann T. (2009). The nanodisc: a novel tool for membrane protein studies. Biol. Chem. 390, 805–814. 10.1515/BC.2009.091 19453280

[B26] Borkowski O. Koch M. Zettor A. Pandi A. Batista A. C. Soudier P. (2020). Large scale active-learning-guided exploration for *in vitro* protein production optimization. Nat. Commun. 11, 1872. 10.1038/s41467-020-15798-5 32312991 PMC7170859

[B27] Boukadida C. Fritz M. Blumen B. Fogeron M. L. Penin F. Martin A. (2018). NS2 proteases from hepatitis C virus and related hepaciviruses share composite active sites and previously unrecognized intrinsic proteolytic activities. Plos Pathog. 14, e1006863. 10.1371/journal.ppat.1006863 29415072 PMC5819835

[B28] Braakman I. Lamriben L. Van Zadelhoff G. Hebert D. N. (2017). Analysis of disulfide bond formation. Curr. Protoc. Protein Sci. 90, 14–21. 10.1002/cpps.43 29091273 PMC5743216

[B29] Brödel A. K. Wüstenhagen D. A. Kubick S. (2015). Cell-free protein synthesis systems derived from cultured mammalian cells. Methods Mol. Biol. 1261, 129–140. 10.1007/978-1-4939-2230-7_7 25502197

[B30] Brown G. R. Hem V. Katz K. S. Ovetsky M. Wallin C. Ermolaeva O. (2015). Gene: a gene-centered information resource at NCBI. Nucleic Acids Res. 43, D36–D42. 10.1093/nar/gku1055 25355515 PMC4383897

[B31] Bundy B. C. Swartz J. R. (2011). Efficient disulfide bond formation in virus-like particles. J. Biotechnol. 154, 230–239. 10.1016/j.jbiotec.2011.04.011 21536082

[B32] Buntru M. Vogel S. Stoff K. Spiegel H. Schillberg S. (2015). A versatile coupled cell-free transcription-translation system based on tobacco BY-2 cell lysates. Biotechnol. Bioeng. 112, 867–878. 10.1002/bit.25502 25421615

[B33] Carraher C. Nazmi A. R. Newcomb R. D. Kralicek A. (2013). Recombinant expression, detergent solubilisation and purification of insect odorant receptor subunits. Protein Expr. Purif. 90, 160–169. 10.1016/j.pep.2013.06.002 23770557

[B34] Chae P. S. Rasmussen S. G. Rana R. R. Gotfryd K. Chandra R. Goren M. A. (2010a). Maltose-neopentyl glycol (MNG) amphiphiles for solubilization, stabilization and crystallization of membrane proteins. Nat. Methods 7, 1003–1008. 10.1038/nmeth.1526 21037590 PMC3063152

[B35] Chae P. S. Rasmussen S. G. Rana R. R. Gotfryd K. Chandra R. Goren M. A. (2010b). Maltose-neopentyl glycol (MNG) amphiphiles for solubilization, stabilization and crystallization of membrane proteins. Nat. Methods 7, 1003–1008. 10.1038/nmeth.1526 21037590 PMC3063152

[B36] Chan P. Thomas C. J. Sprang S. R. Tall G. G. (2013). Molecular chaperoning function of Ric-8 is to fold nascent heterotrimeric G protein α subunits. Proc. Natl. Acad. Sci. U.S.A. 110, 3794–3799. 10.1073/pnas.1220943110 23431197 PMC3593926

[B37] Chen A. Majdinasab E. J. Fiori M. C. Liang H. Altenberg G. A. (2020). Polymer-encased nanodiscs and polymer nanodiscs: new platforms for membrane protein research and applications. Front. Bioeng. Biotechnol. 8, 598450. 10.3389/fbioe.2020.598450 33304891 PMC7701119

[B38] Cole S. D. Miklos A. E. Chiao A. C. Sun Z. Z. Lux M. W. (2020). Methodologies for preparation of prokaryotic extracts for cell-free expression systems. Synth. Syst. Biotechnol. 5, 252–267. 10.1016/j.synbio.2020.07.006 32775710 PMC7398980

[B39] Collaboration O. R. (2016). The ORFeome collaboration: a genome-scale human ORF-clone resource. Nat. Methods 13, 191–192. 10.1038/nmeth.3776 26914201

[B40] Contreras-Llano L. E. Meyer C. Liu Y. Sarker M. Lim S. Longo M. L. (2020). Holistic engineering of cell-free systems through proteome-reprogramming synthetic circuits. Nat. Commun. 11, 3138. 10.1038/s41467-020-16900-7 32561745 PMC7305103

[B41] Coordinators N. R. (2017). Database resources of the national center for biotechnology information. Nucleic Acids Res. 45, D12–D17. 10.1093/nar/gkw1071 27899561 PMC5210554

[B42] Cull M. G. Schatz P. J. (2000). Biotinylation of proteins *in vivo* and *in vitro* using small peptide tags. Meth Enzymol. 326, 430–440. 10.1016/s0076-6879(00)26068-0 11036656

[B43] Dai W. Zhang B. Jiang X. M. Su H. Li J. Zhao Y. (2020). Structure-based design of antiviral drug candidates targeting the SARS-CoV-2 main protease. Science 368, 1331–1335. 10.1126/science.abb4489 32321856 PMC7179937

[B44] Danmaliki G. I. Hwang P. M. (2020). Solution NMR spectroscopy of membrane proteins. Biochim. Biophys. Acta Biomembr. 1862, 183356. 10.1016/j.bbamem.2020.183356 32416193

[B45] David G. Fogeron M. L. Montserret R. Lecoq L. Page A. Delolme F. (2019). Phosphorylation and alternative translation on wheat germ cell-free protein synthesis of the DHBV large envelope protein. Front. Mol. Biosci. 6, 138. 10.3389/fmolb.2019.00138 31850370 PMC6902406

[B46] David G. Fogeron M. L. Schledorn M. Montserret R. Haselmann U. Penzel S. (2018). Structural studies of self-assembled subviral particles: combining cell-free expression with 110 kHz MAS NMR spectroscopy. Angew. Chem. Int. Ed. Engl. 57, 4787–4791. 10.1002/anie.201712091 29457857

[B47] De Hoog H. P. Lin Jierong E. M. Banerjee S. Décaillot F. M. Nallani M. (2014). Conformational antibody binding to a native, cell-free expressed GPCR in block copolymer membranes. PLoS ONE 9, e110847–110847. 10.1371/journal.pone.0110847 25329156 PMC4203850

[B48] Degrip W. J. Vanoostrum J. Bovee-Geurts P. H. (1998). Selective detergent-extraction from mixed detergent/lipid/protein micelles, using cyclodextrin inclusion compounds: a novel generic approach for the preparation of proteoliposomes. Biochem. J. 330 (Pt 2), 667–674. 10.1042/bj3300667 9480873 PMC1219188

[B49] Deniaud A. Liguori L. Blesneac I. Lenormand J. L. Pebay-Peyroula E. (2010). Crystallization of the membrane protein hVDAC1 produced in cell-free system. Biochim. Biophys. Acta 1798, 1540–1546. 10.1016/j.bbamem.2010.04.010 20435015

[B50] Denisov S. S. Ippel J. H. Mans B. J. Dijkgraaf I. Hackeng T. M. (2019). SecScan: a general approach for mapping disulfide bonds in synthetic and recombinant peptides and proteins. Chem. Commun. 55, 1374–1377. 10.1039/c8cc08777f 30520894

[B51] Diederichs S. Bartsch L. Berkmann J. C. Fröse K. Heitmann J. Hoppe C. (2016). The dark matter of the cancer genome: aberrations in regulatory elements, untranslated regions, splice sites, non-coding RNA and synonymous mutations. EMBO Mol. Med. 8, 442–457. 10.15252/emmm.201506055 26992833 PMC5126213

[B52] Dobberstein B. Blobel G. (1977). Functional interaction of plant ribosomes with animal microsomal membranes. Biochem. Biophys. Res. Commun. 74, 1675–1682. 10.1016/0006-291x(77)90637-4 402922

[B53] Dobson C. M. (2019). Biophysical techniques in structural biology. Annu. Rev. Biochem. 88, 25–33. 10.1146/annurev-biochem-013118-111947 30986087

[B54] Dondapati S. K. Stech M. Zemella A. Kubick S. (2020). Cell-free protein synthesis: a promising option for future drug development. BioDrugs 34, 327–348. 10.1007/s40259-020-00417-y 32198631 PMC7211207

[B55] Dürr U. H. N. Gildenberg M. Ramamoorthy A. (2012). The magic of bicelles lights up membrane protein structure. Chem. Rev. 112, 6054–6074. 10.1021/cr300061w 22920148 PMC3497859

[B56] Duyen T. T. Matsuura H. Ujiie K. Muraoka M. Harada K. Hirata K. (2016). Paper-based colorimetric biosensor for antibiotics inhibiting bacterial protein synthesis. J. Biosci. Bioeng. 14, 33–39. 10.1016/j.jbiosc.2016.07.015 27514909

[B57] Einhauer A. Jungbauer A. (2001). The FLAG peptide, a versatile fusion tag for the purification of recombinant proteins. J. Biochem. Biophys. Methods 49, 455–465. 10.1016/s0165-022x(01)00213-5 11694294

[B58] Elbaz Y. Steiner-Mordoch S. Danieli T. Schuldiner S. (2004). *In vitro* synthesis of fully functional EmrE, a multidrug transporter, and study of its oligomeric state. Proc. Natl. Acad. Sci. U.S.A. 101, 1519–1524. 10.1073/pnas.0306533101 14755055 PMC341767

[B59] Ellman G. L. (1959). Tissue sulfhydryl groups. Arch. Biochem. Biophys. 82, 70–77. 10.1016/0003-9861(59)90090-6 13650640

[B60] Endo Y. Otsuzuki S. Ito K. Miura K. (1992). Production of an enzymatic active protein using a continuous flow cell-free translation system. J. Biotechnol. 25, 221–230. 10.1016/0168-1656(92)90157-5 1368801

[B61] Endo Y. Sawasaki T. (2006). Cell-free expression systems for eukaryotic protein production. Curr. Opin. Biotechnol. 17, 373–380. 10.1016/j.copbio.2006.06.009 16828277

[B62] Endo Y. Sawasaki T. (2004). High-throughput, genome-scale protein production method based on the wheat germ cell-free expression system. J. Struct. Funct. Genom. 5, 45–57. 10.1023/B:JSFG.0000029208.83739.49 15263842

[B63] Fan Q. Treder K. Miller W. A. (2012). Untranslated regions of diverse plant viral RNAs vary greatly in translation enhancement efficiency. BMC Biotechnol. 12, 22. 10.1186/1472-6750-12-22 22559081 PMC3416697

[B64] Feige M. J. B. Hendershot L. M. (2018). “Disulfide bonds in protein folding and stability,” in Oxidative folding of proteins: basic principles, cellular regulation and engineering. Editor Feige M. J. (London: Royal Society of Chemistry)), 1–33.

[B65] Fogeron M.-L. Badillo A. Penin F. Böckmann A. (2017). Wheat germ cell-free overexpression for the production of membrane proteins. New York, NY: Springer, 91–108. 10.1007/978-1-4939-7151-0_5 28755365

[B66] Fogeron M. L. Badillo A. Jirasko V. Gouttenoire J. Paul D. Lancien L. (2015a). Wheat germ cell-free expression: two detergents with a low critical micelle concentration allow for production of soluble HCV membrane proteins. Protein Expr. Purif. 105, 39–46. 10.1016/j.pep.2014.10.003 25306874

[B67] Fogeron M. L. Badillo A. Penin F. Böckmann A. (2017). Wheat germ cell-free overexpression for the production of membrane proteins. Methods Mol. Biol. 1635, 91–108. 10.1007/978-1-4939-7151-0_5 28755365

[B68] Fogeron M. L. Jirasko V. Penzel S. Paul D. Montserret R. Danis C. (2016). Cell-free expression, purification, and membrane reconstitution for NMR studies of the nonstructural protein 4B from hepatitis C virus. J. Biomol. NMR 65, 87–98. 10.1007/s10858-016-0040-2 27233794

[B69] Fogeron M. L. Paul D. Jirasko V. Montserret R. Lacabanne D. Molle J. (2015b). Functional expression, purification, characterization, and membrane reconstitution of non-structural protein 2 from hepatitis C virus. Protein Expr. Purif. 116, 1–6. 10.1016/j.pep.2015.08.027 26325423

[B70] Gad W. Nair M. G. Van Belle K. Wahni K. De Greve H. Van Ginderachter J. A. (2013). The quiescin sulfhydryl oxidase (hQSOX1b) tunes the expression of resistin-like molecule alpha (RELM-α or mFIZZ1) in a wheat germ cell-free extract. PLoS ONE 8, e55621. 10.1371/journal.pone.0055621 23383248 PMC3561318

[B71] Gagoski D. Mureev S. Giles N. Johnston W. Dahmer-Heath M. Škalamera D. (2015). Gateway-compatible vectors for high-throughput protein expression in pro- and eukaryotic cell-free systems. J. Biotechnol. 195, 1–7. 10.1016/j.jbiotec.2014.12.006 25529348

[B72] Gan R. Jewett M. C. (2014). A combined cell-free transcription-translation system from *Saccharomyces cerevisiae* for rapid and robust protein synthe. Biotechnol. J. 9, 641–651. 10.1002/biot.201300545 24677809

[B73] Gao X. Dong X. Li X. Liu Z. Liu H. (2020). Prediction of disulfide bond engineering sites using a machine learning method. Sci. Rep. 10, 10330. 10.1038/s41598-020-67230-z 32587353 PMC7316719

[B74] Garavito R. M. Ferguson-Miller S. (2001). Detergents as tools in membrane biochemistry. J. Biol. Chem. 276, 32403–32406. 10.1074/jbc.R100031200 11432878

[B75] Geertz M. Shore D. Maerkl S. J. (2012). Massively parallel measurements of molecular interaction kinetics on a microfluidic platform. Proc. Natl. Acad. Sci. U.S.A. 109, 16540–16545. 10.1073/pnas.1206011109 23012409 PMC3478601

[B76] Genji T. Nozawa A. Tozawa Y. (2010). Efficient production and purification of functional bacteriorhodopsin with a wheat-germ cell-free system and a combination of Fos-choline and CHAPS detergents. Biochem. Biophys. Res. Commun. 400, 638–642. 10.1016/j.bbrc.2010.08.119 20807510

[B77] Gibson D. G. Young L. Chuang R. Y. Venter J. C. Hutchison C. A. 3rd Smith H. O. (2009). Enzymatic assembly of DNA molecules up to several hundred kilobases. Nat. Methods 6, 343–345. 10.1038/nmeth.1318 19363495

[B78] Goren M. A. Fox B. G. (2008). Wheat germ cell-free translation, purification, and assembly of a functional human stearoyl-CoA desaturase complex. Protein Expr. Purif. 62, 171–178. 10.1016/j.pep.2008.08.002 18765284 PMC2586813

[B79] Goren M. A. Nozawa A. Makino S. Wrobel R. L. Fox B. G. (2009). Cell-free translation of integral membrane proteins into unilamelar liposomes. Meth Enzymol. 463, 647–673. 10.1016/S0076-6879(09)63037-8 PMC5814320 19892197

[B80] Goshima N. Kawamura Y. Fukumoto A. Miura A. Honma R. Satoh R. (2008). Human protein factory for converting the transcriptome into an in vitro-expressed proteome,. Nat. Methods 5, 1011–1017. 10.1038/nmeth.1273 19054851

[B81] Gräwe A. Dreyer A. Vornholt T. Barteczko U. Buchholz L. Drews G. (2019). A paper-based, cell-free biosensor system for the detection of heavy metals and date rape drugs. PLoS One 14, e0210940. 10.1371/journal.pone.0210940 30840628 PMC6402643

[B82] Gregorio N. E. Levine M. Z. Oza J. P. (2019). A user’s guide to cell-free protein synthesis. Methods Protoc. 2. 10.3390/mps2010024 PMC6481089 31164605

[B83] Guild K. Zhang Y. Stacy R. Mundt E. Benbow S. Green A. (2011). Wheat germ cell-free expression system as a pathway to improve protein yield and solubility for the SSGCID pipeline. Acta Crystallogr. Sect F Struct. Biol. Cryst. Commun. 67, 1027–1031. 10.1107/S1744309111032143 PMC3169397 21904045

[B84] Guo C. Williams J. C. Polenova T. (2019). Conformational flexibility of p150Glued(1-191) subunit of dynactin assembled with microtubules. Biophys. J. 117, 938–949. 10.1016/j.bpj.2019.07.036 31445682 PMC6731383

[B85] Gustafsson C. Minshull J. Govindarajan S. Ness J. Villalobos A. Welch M. (2012). Engineering genes for predictable protein expression. Protein Expr. Purif. 83, 37–46. 10.1016/j.pep.2012.02.013 22425659 PMC3746766

[B86] Haberstock S. Roos C. Hoevels Y. Dötsch V. Schnapp G. Pautsch A. (2012). A systematic approach to increase the efficiency of membrane protein production in cell-free expression systems. Protein Expr. Purif. 82, 308–316. 10.1016/j.pep.2012.01.018 22342679

[B87] Hagn F. Etzkorn M. Raschle T. Wagner G. (2013). Optimized phospholipid bilayer nanodiscs facilitate high-resolution structure determination of membrane proteins. J. Am. Chem. Soc. 135, 1919–1925. 10.1021/ja310901f 23294159 PMC3566289

[B88] Harayama T. Riezman H. (2018). Understanding the diversity of membrane lipid composition. Nat. Rev. Mol. Cel Biol 19, 281–296. 10.1038/nrm.2017.138 29410529

[B89] Harbers M. (2008). The current status of cDNA cloning. Genomics 91, 232–242. 10.1016/j.ygeno.2007.11.004 18222633

[B90] Harbers M. (2014). Wheat germ systems for cell-free protein expression. FEBS Lett. 588, 2762–2773. 10.1016/j.febslet.2014.05.061 24931374

[B91] Heindl D. D. Hoffmann T. Metzler T. D. Mutter W. D. Nemetz C. D. Watzele M. D. (2002). Method for protein expression starting from stabilized linear short DNA in cell-free in vitro transcription/translation systems with exonuclease-containing lysates or in a cellular system containing exonucleases.Google Patents.

[B92] Henrich E. Dötsch V. Bernhard F. (2015). Screening for lipid requirements of membrane proteins by combining cell-free expression with nanodiscs. Meth Enzymol. 556, 351–369. 10.1016/bs.mie.2014.12.016 25857790

[B93] Hershewe J. Kightlinger W. Jewett M. C. (2020). Cell-free systems for accelerating glycoprotein expression and biomanufacturing. J. Ind. Microbiol. Biotechnol. 12, 121–129. 10.20944/preprints202003.0461.v2 PMC7578589 33090335

[B94] Hibi K. Amikura K. Sugiura N. Masuda K. Ohno S. Yokogawa T. (2020). Reconstituted cell-free protein synthesis using *in vitro* transcribed tRNAs. Commun. Biol. 3, 350. 10.1038/s42003-020-1074-2 32620935 PMC7334211

[B95] Hodgman C. E. Jewett M. C. (2014). Characterizing IGR IRES-mediated translation initiation for use in yeast cell-free protein synthesis. N. Biotechnol. 31, 499–505. 10.1016/j.nbt.2014.07.001 25017988

[B96] Hong S. H. Kwon Y. C. Jewett M. C. (2014). Non-standard amino acid incorporation into proteins using *Escherichia coli* cell-free protein synthesis. Front. Chem. 2, 34. 10.3389/fchem.2014.00034 24959531 PMC4050362

[B97] Hopkins A. L. Groom C. R. (2002). The druggable genome. Nat. Rev. Drug Discov. 1, 727–730. 10.1038/nrd892 12209152

[B98] Inaba K. Masui S. Iida H. Vavassori S. Sitia R. Suzuki M. (2010). Crystal structures of human Ero1α reveal the mechanisms of regulated and targeted oxidation of PDI. EMBO J. 29, 3330–3343. 10.1038/emboj.2010.222 20834232 PMC2957217

[B99] Ishihara G. Goto M. Saeki M. Ito K. Hori T. Kigawa T. (2005). Expression of G protein coupled receptors in a cell-free translational system using detergents and thioredoxin-fusion vectors. Protein Expr. Purif. 41, 27–37. 10.1016/j.pep.2005.01.013 15802218

[B100] Jackson K. Jin S. Fan Z. H. (2015). Optimization of a miniaturized fluid array device for cell-free protein synthesis. Biotechnol. Bioeng. 112, 2459–2467. 10.1002/bit.25668 26037852

[B101] Jackson K. Khnouf R. Fan Z. H. (2014). Cell-free protein synthesis in microfluidic 96-well plates. Methods Mol. Biol. 1118, 157–168. 10.1007/978-1-62703-782-2_10 24395415

[B102] Jackson R. C. Blobel G. (1977). Post-translational cleavage of presecretory proteins with an extract of rough microsomes from dog pancreas containing signal peptidase activity. Proc. Natl. Acad. Sci. U.S.A. 74, 5598–5602. 10.1073/pnas.74.12.5598 271987 PMC431824

[B103] Jarecki B. W. Makino S. Beebe E. T. Fox B. G. Chanda B. (2013). Function of Shaker potassium channels produced by cell-free translation upon injection into Xenopus oocytes. Sci. Rep. 3, 1040. 10.1038/srep01040 23301161 PMC3539143

[B104] Jin Z. Du X. Xu Y. Deng Y. Liu M. Zhao Y. (2020). Structure of Mpro from SARS-CoV-2 and discovery of its inhibitors. Nature 582, 289–293. 10.1038/s41586-020-2223-y 32272481

[B105] Jirasko V. Lakomek N. A. Penzel S. Fogeron M. L. Bartenschlager R. Meier B. H. (2020). Proton-detected solid-state NMR of the cell-free synthesized α-helical transmembrane protein NS4B from hepatitis C virus. Chembiochem 21, 1453–1460. 10.1002/cbic.201900765 31850615 PMC7318649

[B106] Jung J. K. Alam K. K. Verosloff M. S. Capdevila D. A. Desmau M. Clauer P. R. (2020). Cell-free biosensors for rapid detection of water contaminants. Nat. Biotechnol. 13, 29. 10.1016/j.bios.2009.03.001 PMC7718425 32632301

[B107] Kaiser L. Graveland-Bikker J. Steuerwald D. Vanberghem M. Herlihy K. Zhang S. (2008). Efficient cell-free production of olfactory receptors: detergent optimization, structure, and ligand binding analyses. Proc. Natl. Acad. Sci. USA 105, 15726–15731. 10.1073/pnas.0804766105 18840687 PMC2572932

[B108] Kameda A. Morita E. H. Sakurai K. Naiki H. Goto Y. (2009). NMR-based characterization of a refolding intermediate of beta2-microglobulin labeled using a wheat germ cell-free system. Protein Sci. 18, 1592–1601. 10.1002/pro.179 19606503 PMC2776947

[B109] Kamura N. Sawasaki T. Kasahara Y. Takai K. Endo Y. (2005). Selection of 5'-untranslated sequences that enhance initiation of translation in a cell-free protein synthesis system from wheat embryos. Bioorg. Med. Chem. Lett. 15, 5402–5406. 10.1016/j.bmcl.2005.09.013 16213724

[B110] Kanoi B. N. Nagaoka H. Morita M. Tsuboi T. Takashima E. (2021). Leveraging the wheat germ cell-free protein synthesis system to accelerate malaria vaccine development. Parasitol. Int. 80, 102224. 10.1016/j.parint.2020.102224 33137499

[B111] Kanoi B. N. Takashima E. Morita M. White M. T. Palacpac N. M. Ntege E. H. (2017). Antibody profiles to wheat germ cell-free system synthesized Plasmodium falciparum proteins correlate with protection from symptomatic malaria in Uganda. Vaccine 35, 873–881. 10.1016/j.vaccine.2017.01.001 28089547

[B112] Karim A. S. Dudley Q. M. Juminaga A. Yuan Y. Crowe S. A. Heggestad J. T. (2020). *In vitro* prototyping and rapid optimization of biosynthetic enzymes for cell design. Nat. Chem. Biol. 13, 113. 10.1007/978-1-4615-5263-5_2 32541965

[B113] Katz F. N. Rothman J. E. Lingappa V. R. Blobel G. Lodish H. F. (1977). Membrane assembly *in vitro*: synthesis, glycosylation, and asymmetric insertion of a transmembrane protein. Proc. Natl. Acad. Sci. U.S.A. 74, 3278–3282. 10.1073/pnas.74.8.3278 198778 PMC431530

[B114] Katzen F. Chang G. Kudlicki W. (2005). The past, present and future of cell-free protein synthesis. Trends Biotechnol. 23, 150–156. 10.1016/j.tibtech.2005.01.003 15734558

[B115] Kaur H. Abreu B. Akhmetzyanov D. Lakatos-Karoly A. Soares C. M. Prisner T. (2018). Unexplored nucleotide binding modes for the ABC exporter MsbA. J. Am. Chem. Soc. 140, 14112–14125. 10.1021/jacs.8b06739 30289253

[B116] Kaur H. Lakatos A. Spadaccini R. Vogel R. Hoffmann C. Becker-Baldus J. (2015). The ABC exporter MsbA probed by solid state NMR – challenges and opportunities. Biol. Chem. 396, 1135–1149. 10.1515/hsz-2015-0119 25849794 PMC7906285

[B117] Kaur H. Lakatos-Karoly A. Vogel R. Nöll A. Tampé R. Glaubitz C. (2016). Coupled ATPase-adenylate kinase activity in ABC transporters. Nat. Commun. 7, 13864. 10.1038/ncomms13864 28004795 PMC5192220

[B118] Kaur J. Kriebel C. N. Eberhardt P. Jakdetchai O. Leeder A. J. Weber I. (2019). Solid-state NMR analysis of the sodium pump Krokinobacter rhodopsin 2 and its H30A mutant. J. Struct. Biol. 206, 55–65. 10.1016/j.jsb.2018.06.001 29879487

[B119] Kawasaki T. Gouda M. D. Sawasaki T. Takai K. Endo Y. (2003). Efficient synthesis of a disulfide-containing protein through a batch cell-free system from wheat germ. Eur. J. Biochem. 270, 4780–4786. 10.1046/j.1432-1033.2003.03880.x 14622267

[B120] Kelly S. M. Jess T. J. Price N. C. (2005). How to study proteins by circular dichroism. Biochim. Biophys. Acta 1751, 119–139. 10.1016/j.bbapap.2005.06.005 16027053

[B121] Khan F. He M. Taussig M. J. (2006). Double-hexahistidine tag with high-affinity binding for protein immobilization, purification, and detection on ni-nitrilotriacetic acid surfaces. Anal. Chem. 78, 3072–3079. 10.1021/ac060184l 16642995

[B122] Khnouf R. Olivero D. Jin S. Coleman M. A. Fan Z. H. (2010). Cell-free expression of soluble and membrane proteins in an array device for drug screening. Anal. Chem. 82, 7021–7026. 10.1021/ac1015479 20666430

[B123] Ki M. R. Pack S. P. (2020). Fusion tags to enhance heterologous protein expression. Appl. Microbiol. Biotechnol. 104, 2411–2425. 10.1007/s00253-020-10402-8 31993706

[B124] Kido K. Yamanaka S. Nakano S. Motani K. Shinohara S. Nozawa A. (2020). AirID, a novel proximity biotinylation enzyme, for analysis of protein-protein interactions. Elife 9, 113. 10.7554/eLife.54983 PMC7302878 32391793

[B125] Kightlinger W. Warfel K. F. Delisa M. P. Jewett M. C. (2020). Synthetic glycobiology: parts, systems, and applications. ACS Synth. Biol. 9, 1534–1562. 10.1021/acssynbio.0c00210 32526139 PMC7372563

[B126] Kim D. M. Swartz J. R. (2003). Efficient production of a bioactive, multiple disulfide-bonded protein using modified extracts of *Escherichia coli* . Biotechnol. Bioeng. 85, 122–129. 10.1002/bit.10865 14704994

[B127] Kim H. C. Kim K. S. Kang T. J. Choi J. H. Song J. J. Choi Y. H. (2015). Implementing bacterial acid resistance into cell-free protein synthesis for buffer-free expression and screening of enzymes. Biotechnol. Bioeng. 112, 2630–2635. 10.1002/bit.25671 26059009

[B128] Kimple M. E. Brill A. L. Pasker R. L. (2013). Overview of affinity tags for protein purification. Curr. Protoc. Protein Sci. 73, 9. 10.1002/0471140864.ps0909s73 PMC4527311 24510596

[B129] Klammt C. Löhr F. Schäfer B. Haase W. Dötsch V. Rüterjans H. (2004). High level cell-free expression and specific labeling of integral membrane proteins. Eur. J. Biochem. 271, 568–580. 10.1111/j.1432-1033.2003.03959.x 14728684

[B130] Klammt C. Srivastava A. Eifler N. Junge F. Beyermann M. Schwarz D. (2007). Functional analysis of cell-free-produced human endothelin B receptor reveals transmembrane segment 1 as an essential area for ET-1 binding and homodimer formation. FEBS J. 274, 3257–3269. 10.1111/j.1742-4658.2007.05854.x 17535295

[B131] Klammt C. Schwarz D. Fendler K. Haase W. Dötsch V. Bernhard F. (2005). Evaluation of detergents for the soluble expression of α-helical and β-barrel-type integral membrane proteins by a preparative scale individual cell-free expression system. FEBS J. 272, 6024–6038. 10.1111/j.1742-4658.2005.05002.x 16302967

[B132] Kobilka B. K. (1990). The role of cytosolic and membrane factors in processing of the human beta-2 adrenergic receptor following translocation and glycosylation in a cell-free system. J. Biol. Chem. 265, 7610–7618. 10.1016/s0021-9258(19)39158-6 1692024

[B133] Kögler L. M. Stichel J. Beck-Sickinger A. G. (2019). Structural investigations of cell-free expressed G protein-coupled receptors. Biol. Chem. 401, 97–116. 10.1515/hsz-2019-0292 31539345

[B134] Kohno T. Endo Y. (2007). Production of protein for nuclear magnetic resonance study using the wheat germ cell-free system. Methods Mol. Biol. 375, 257–272. 10.1007/978-1-59745-388-2_13 17634606

[B135] Kohno T. (2010). NMR assignment method for amide signals with cell-free protein synthesis system. Methods Mol. Biol. 607, 113–126. 10.1007/978-1-60327-331-2_11 20204853

[B136] Kralicek A. (2014). A cell-free expression screen to identify fusion tags for improved protein expression. Methods Mol. Biol. 1118, 35–54. 10.1007/978-1-62703-782-2_3 24395408

[B137] Krug U. Gloge A. Schmidt P. Becker-Baldus J. Bernhard F. Kaiser A. (2020). The conformational equilibrium of the neuropeptide Y2 receptor in bilayer membranes. Angew. Chem. Int. Ed. Engl. 59, 23854–23861. 10.1002/anie.202006075 32790043 PMC7736470

[B138] Lacabanne D. Fogeron M. L. Wiegand T. Cadalbert R. Meier B. H. Böckmann A. (2019). Protein sample preparation for solid-state NMR investigations. Prog. Nucl. Magn. Reson. Spectrosc. 110, 20–33. 10.1016/j.pnmrs.2019.01.001 30803692

[B139] Lacabanne D. Lends A. Danis C. Kunert B. Fogeron M. L. Jirasko V. (2017). Gradient reconstitution of membrane proteins for solid-state NMR studies. J. Biomol. NMR 69, 81–91. 10.1007/s10858-017-0135-4 28900789

[B140] Ladizhansky V. (2017). Applications of solid-state NMR to membrane proteins. Biochim. Biophys. Acta 1865, 1577–1586. 10.1016/j.bbapap.2017.07.004 28709996

[B141] Lakbub J. C. Shipman J. T. Desaire H. (2018). Recent mass spectrometry-based techniques and considerations for disulfide bond characterization in proteins. Anal. Bioanal. Chem. 410, 2467–2484. 10.1007/s00216-017-0772-1 29256076 PMC5857437

[B142] Laohakunakorn N. (2020). Cell-free systems: a proving ground for rational biodesign. Front. Bioeng. Biotechnol. 8, 788. 10.3389/fbioe.2020.00788 32793570 PMC7393481

[B143] Lecoq L. Schledorn M. Wang S. Smith-Penzel S. Malär A. A. Callon M. (2019). 100 kHz MAS proton-detected NMR spectroscopy of hepatitis B virus capsids. Front. Mol. Biosci. 6, 58. 10.3389/fmolb.2019.00058 31396521 PMC6668038

[B144] Lecoq L. Wang S. Wiegand T. Bressanelli S. Nassal M. Meier B. H. (2018). Localizing conformational hinges by NMR: where do hepatitis B virus core proteins adapt for capsid assembly? Chem. Phys. Chem. 19, 1336–1340. 10.1002/cphc.201800211 29542854

[B145] Li B. Makino S. Beebe E. T. Urano D. Aceti D. J. Misenheimer T. M. (2016). Cell-free translation and purification of *Arabidopsis thaliana* regulator of G signaling 1 protein. Protein Expr. Purif. 126, 33–41. 10.1016/j.pep.2016.04.016 27164033 PMC5594927

[B146] Li K. Jiang Q. Bai X. Yang Y. F. Ruan M. Y. Cai S. Q. (2017). Tetrameric assembly of K+ channels requires ER-located chaperone proteins. Mol. Cel. 65, 52–65. 10.1016/j.molcel.2016.10.027 27916661

[B147] Lingappa J. R. Newman M. A. Klein K. C. Dooher J. E. (2005). Comparing capsid assembly of primate lentiviruses and hepatitis B virus using cell-free systems. Virology 333, 114–123. 10.1016/j.virol.2004.12.024 15708597

[B148] Lingappa V. R. Lingappa J. R. Prasad R. Ebner K. E. Blobel G. (1978). Coupled cell-free synthesis, segregation, and core glycosylation of a secretory protein. Proc. Natl. Acad. Sci. U.S.A. 75, 2338–2342. 10.1073/pnas.75.5.2338 276877 PMC392548

[B149] Long A. R. O'Brien C. C. Alder N. N. (2012). The cell-free integration of a polytopic mitochondrial membrane protein into liposomes occurs cotranslationally and in a lipid-dependent manner. PLoS ONE 7, e46332. 10.1371/journal.pone.0046332 23050015 PMC3457961

[B150] Los G. V. Encell L. P. Mcdougall M. G. Hartzell D. D. Karassina N. Zimprich C. (2008). HaloTag: a novel protein labeling technology for cell imaging and protein analysis. ACS Chem. Biol. 3, 373–382. 10.1021/cb800025k 18533659

[B151] Lyford L. K. Rosenberg R. L. (1999). Cell-free expression and functional reconstitution of homo-oligomeric alpha7 nicotinic acetylcholine receptors into planar lipid bilayers. J. Biol. Chem. 274, 25675–25681. 10.1074/jbc.274.36.25675 10464304

[B152] Lyukmanova E. N. Shenkarev Z. O. Khabibullina N. F. Kopeina G. S. Shulepko M. A. Paramonov A. S. (2012a). Lipid-protein nanodiscs for cell-free production of integral membrane proteins in a soluble and folded state: comparison with detergent micelles, bicelles and liposomes. Biochim. Biophys. Acta 1818, 349–358. 10.1016/j.bbamem.2011.10.020 22056981

[B153] Lyukmanova E. N. Shenkarev Z. O. Khabibullina N. F. Kulbatskiy D. S. Shulepko M. A. Petrovskaya L. E. (2012b). N-terminal fusion tags for effective production of g-protein-coupled receptors in bacterial cell-free systems. Acta Naturae 4, 58–64. 10.32607/20758251-2012-4-4-58-64 23346380 PMC3548174

[B154] Madin K. Sawasaki T. Ogasawara T. Endo Y. (2000). A highly efficient and robust cell-free protein synthesis system prepared from wheat embryos: plants apparently contain a suicide system directed at ribosomes. Proc. Natl. Acad. Sci. U.S.A. 97, 559–564. 10.1073/pnas.97.2.559 10639118 PMC15369

[B155] Maertens B. Spriestersbach A. Kubicek J. Schäfer F. (2015). Strep-tagged protein purification. Meth Enzymol. 559, 53–69. 10.1016/bs.mie.2014.11.008 26096503

[B156] Makino S. Beebe E. T. Markley J. L. Fox B. G. (2014). Cell-free protein synthesis for functional and structural studies. Methods Mol. Biol. 1091, 161–178. 10.1007/978-1-62703-691-7_11 24203331

[B157] Makino S. Goren M. A. Fox B. G. Markley J. L. (2010). Cell-free protein synthesis technology in NMR high-throughput structure determination. Methods Mol. Biol. 607, 127–147. 10.1007/978-1-60327-331-2_12 20204854

[B158] Malhotra A. (2009). Tagging for protein expression. Meth Enzymol. 463, 239–258. 10.1016/S0076-6879(09)63016-0 19892176

[B159] Malhotra K. Alder N. N. (2017). Reconstitution of mitochondrial membrane proteins into nanodiscs by cell-free expression. Methods Mol. Biol. 1567, 155–178. 10.1007/978-1-4939-6824-4_10 28276018 PMC5663215

[B160] Martin R. W. Des Soye B. J. Kwon Y. C. Kay J. Davis R. G. Thomas P. M. (2018). Cell-free protein synthesis from genomically recoded bacteria enables multisite incorporation of noncanonical amino acids. Nat. Commun. 9, 1203. 10.1038/s41467-018-03469-5 29572528 PMC5865108

[B161] Matsuba I. Hatori N. Koido N. Watanabe Y. Ebara F. Matsuzawa Y. (2020). Survey of the current status of subclinical coronavirus disease 2019 (COVID-19). J. Infect. Chemother. 26, 1294–1300. 10.1016/j.jiac.2020.09.005 32958395 PMC7474902

[B162] Matsumoto M. Matsuzaki F. Oshikawa K. Goshima N. Mori M. Kawamura Y. (2017). A large-scale targeted proteomics assay resource based on an *in vitro* human proteome. Nat. Methods 14, 251–258. 10.1038/nmeth.4116 28267743

[B163] Matsunaga S. Kawakami S. Matsuo I. Okayama A. Tsukagoshi H. Kudoh A. (2014). Wheat germ cell-free system-based production of hemagglutinin-neuraminidase glycoprotein of human parainfluenza virus type 3 for generation and characterization of monoclonal antibody. Front. Microbiol. 5, 208. 10.3389/fmicb.2014.00208 24860558 PMC4026691

[B164] Matsuoka K. Komori H. Nose M. Endo Y. Sawasaki T. (2010). Simple screening method for autoantigen proteins using the N-terminal biotinylated protein library produced by wheat cell-free synthesis. J. Proteome Res. 9, 4264–4273. 10.1021/pr9010553 20575507 PMC2917173

[B165] Mcallister W. T. Raskin C. A. (1993). The phage RNA polymerases are related to DNA polymerases and reverse transcriptases. Mol. Microbiol. 10, 1–6. 10.1111/j.1365-2958.1993.tb00897.x 7526118

[B166] Michel E. Wüthrich K. (2012). Cell-free expression of disulfide-containing eukaryotic proteins for structural biology. FEBS J. 279, 3176–3184. 10.1111/j.1742-4658.2012.08697.x 22776321

[B167] Mikami S. Kobayashi T. Masutani M. Yokoyama S. Imataka H. (2008). A human cell-derived *in vitro* coupled transcription/translation system optimized for production of recombinant proteins. Protein Expr. Purif. 62, 190–198. 10.1016/j.pep.2008.09.002 18814849

[B168] Minkoff B. B. Makino S. I. Haruta M. Beebe E. T. Wrobel R. L. Fox B. G. (2017). A cell-free method for expressing and reconstituting membrane proteins enables functional characterization of the plant receptor-like protein kinase FERONIA. J. Biol. Chem. 292, 5932–5942. 10.1074/jbc.M116.761981 28235802 PMC5392584

[B169] Miot M. Betton J. M. (2011). Reconstitution of the Cpx signaling system from cell-free synthesized proteins. N. Biotechnol. 28, 277–281. 10.1016/j.nbt.2010.06.012 20601270

[B170] Miyazono K. Watanabe M. Kosinski J. Ishikawa K. Kamo M. Sawasaki T. (2007). Novel protein fold discovered in the PabI family of restriction enzymes. Nucleic Acids Res. 35, 1908–1918. 10.1093/nar/gkm091 17332011 PMC1874622

[B171] Mohr B. P. Retterer S. T. Doktycz M. J. (2016). While-you-wait proteins? Producing biomolecules at the point of need. Expert Rev. Proteomics 13, 707–709. 10.1080/14789450.2016.1209415 27402489

[B172] Morishita R. Sugiyama S. Denda M. Tokunaga S. Kido K. Shioya R. (2019). CF-PA2Vtech: a cell-free human protein array technology for antibody validation against human proteins. Sci. Rep. 9, 19349. 10.1038/s41598-019-55785-5 31852950 PMC6920144

[B173] Morita E. H. Sawasaki T. Tanaka R. Endo Y. Kohno T. (2003). A wheat germ cell-free system is a novel way to screen protein folding and function. Protein Sci. 12, 1216–1221. 10.1110/ps.0241203 12761392 PMC2323893

[B174] Morita E. H. Shimizu M. Ogasawara T. Endo Y. Tanaka R. Kohno T. (2004). A novel way of amino acid-specific assignment in (1)H-(15)N HSQC spectra with a wheat germ cell-free protein synthesis system. J. Biomol. NMR 30, 37–45. 10.1023/B:JNMR.0000042956.65678.b8 15452433

[B175] Morita M. Takashima E. Ito D. Miura K. Thongkukiatkul A. Diouf A. (2017). Immunoscreening of Plasmodium falciparum proteins expressed in a wheat germ cell-free system reveals a novel malaria vaccine candidate. Sci. Rep. 7, 46086. 10.1038/srep46086 28378857 PMC5380959

[B176] Nagy J. K. Kuhn Hoffmann A. Keyes M. H. Gray D. N. Oxenoid K. Sanders C. R. (2001). Use of amphipathic polymers to deliver a membrane protein to lipid bilayers. FEBS Lett. 501, 115–120. 10.1016/s0014-5793(01)02627-8 11470268

[B177] Nagy S. K. Kállai B. M. András J. Mészáros T. (2020). A novel family of expression vectors with multiple affinity tags for wheat germ cell- free protein expression, Berlin: Springer, 1–9. 10.1186/s12896-020-00610-5 PMC7071761 32169064

[B178] Nemoto K. Kagawa M. Nozawa A. Hasegawa Y. Hayashi M. Imai K. (2018). Identification of new abscisic acid receptor agonists using a wheat cell-free based drug screening system. Sci. Rep. 8, 4268. 10.1038/s41598-018-22538-9 29523814 PMC5844987

[B179] Niwa T. Kanamori T. Ueda T. Taguchi H. (2012). Global analysis of chaperone effects using a reconstituted cell-free translation system. Proc. Natl. Acad. Sci. U.S.A. 109, 8937–8942. 10.1073/pnas.1201380109 22615364 PMC3384135

[B180] Nomura S. M. Kondoh S. Asayama W. Asada A. Nishikawa S. Akiyoshi K. (2008). Direct preparation of giant proteo-liposomes by *in vitro* membrane protein synthesis. J. Biotechnol. 133, 190–195. 10.1016/j.jbiotec.2007.08.023 17900734

[B181] Novikova I. V. Sharma N. Moser T. Sontag R. Liu Y. Collazo M. J. (2018). Protein structural biology using cell-free platform from wheat germ. Adv. Struct. Chem. Imaging 4, 13. 10.1186/s40679-018-0062-9 30524935 PMC6244559

[B182] Nozawa A. Nanamiya H. Miyata T. Linka N. Endo Y. Weber A. P. (2007). A cell-free translation and proteoliposome reconstitution system for functional analysis of plant solute transporters. Plant Cel. Physiol. 48, 1815–1820. 10.1093/pcp/pcm150 17981875

[B183] Nozawa A. Ogasawara T. Matsunaga S. Iwasaki T. Sawasaki T. Endo Y. (2011). Production and partial purification of membrane proteins using a liposome-supplemented wheat cell-free translation system. BMC Biotechnol. 11, 35. 10.1186/1472-6750-11-35 21481249 PMC3090341

[B184] Nozawa A. Tozawa Y. (2014a). Incorporation of adenine nucleotide transporter, Ant1p, into proteoliposomes facilitates ATP translocation and activation of encapsulated luciferase. J. Biosci. Bioeng. 118, 130–133. 10.1016/j.jbiosc.2014.02.001 24656877

[B185] Nozawa A. Tozawa Y. (2014b). Modifications of wheat germ cell-free system for functional proteomics of plant membrane proteins. Methods Mol. Biol. 1072, 259–272. 10.1007/978-1-62703-631-3_19 24136528

[B186] Ogawa A. Tabuchi J. Doi Y. (2014). Identification of short untranslated regions that sufficiently enhance translation in high-quality wheat germ extract. Bioorg. Med. Chem. Lett. 24, 3724–3727. 10.1016/j.bmcl.2014.07.004 25037913

[B187] Ogawa A. Tabuchi J. Doi Y. Takamatsu M. (2016). Biofunction-assisted DNA detection through RNase H-enhanced 3' processing of a premature tRNA probe in a wheat germ extract. Bioorg. Med. Chem. Lett. 26, 3658–3661. 10.1016/j.bmcl.2016.05.091 27289318

[B188] Okada K. Muneyoshi Y. Endo Y. Hori H. (2009). Production of yeast (m2G10) methyltransferase (Trm11 and Trm112 complex) in a wheat germ cell-free translation system. Nucleic Acids Symp. Ser. 14, 303–304. 10.1093/nass/nrp152 19749381

[B189] Okimune K. I. Nagy S. K. Hataya S. Endo Y. Takasuka T. E. (2020). Reconstitution of Drosophila and human chromatins by wheat germ cell-free co-expression system. BMC Biotechnol. 20, 62. 10.1186/s12896-020-00655-6 33261588 PMC7708258

[B190] Ong Y. S. Lakatos A. Becker-Baldus J. Pos K. M. Glaubitz C. (2013). Detecting substrates bound to the secondary multidrug efflux pump EmrE by DNP-enhanced solid-state NMR. J. Am. Chem. Soc. 135, 15754–15762. 10.1021/ja402605s 24047229

[B191] Oza J. P. Aerni H. R. Pirman N. L. Barber K. W. Ter Haar C. M. Rogulina S. (2015). Robust production of recombinant phosphoproteins using cell-free protein synthesis. Nat. Commun. 6, 8168. 10.1038/ncomms9168 26350765 PMC4566161

[B192] Pacull E. M. Sendker F. Bernhard F. Scheidt H. A. Schmidt P. Huster D. (2020). Integration of cell-free expression and solid-state NMR to investigate the dynamic properties of different sites of the Growth Hormone secretagogue receptor. Front. Pharmacol. 11, 562113. 10.3389/fphar.2020.562113 33324203 PMC7723455

[B193] Pardee K. Green A. A. Ferrante T. Cameron D. E. Daleykeyser A. Yin P. (2014). Paper-based synthetic gene networks. Cell 159, 940–954. 10.1016/j.cell.2014.10.004 25417167 PMC4243060

[B194] Park K. H. Berrier C. Lebaupain F. Pucci B. Popot J. L. Ghazi A. (2007). Fluorinated and hemifluorinated surfactants as alternatives to detergents for membrane protein cell-free synthesis. Biochem. J. 403, 183. 10.1042/BJ20061473 17176254 PMC1828882

[B195] Park K. H. Billon-Denis E. Dahmane T. Lebaupain F. Pucci B. Breyton C. (2011). In the cauldron of cell-free synthesis of membrane proteins: playing with new surfactants. N. Biotechnol. 28, 255–261. 10.1016/j.nbt.2010.08.008 20800706

[B196] Perez J. G. Stark J. C. Jewett M. C. (2016). Cell-free synthetic biology: engineering beyond the cell. Cold Spring Harb Perspect. Biol. 8. 121. 10.1101/cshperspect.a023853 PMC5131772 27742731

[B197] Periasamy A. Shadiac N. Amalraj A. Garajová S. Nagarajan Y. Waters S. (2013). Cell-free protein synthesis of membrane (1,3)-β-d-glucan (curdlan) synthase: co-translational insertion in liposomes and reconstitution in nanodiscs. Biochim. Biophys. Acta 1828, 743–757. 10.1016/j.bbamem.2012.10.003 23063656

[B198] Popot J. L. (2010). Amphipols, nanodiscs, and fluorinated surfactants: three nonconventional approaches to studying membrane proteins in aqueous solutions. Annu. Rev. Biochem. 79, 737–775. 10.1146/annurev.biochem.052208.114057 20307193

[B199] Punta M. Forrest L. R. Bigelow H. Kernytsky A. Liu J. Rost B. (2007). Membrane protein prediction methods. Methods 41, 460–474. 10.1016/j.ymeth.2006.07.026 17367718 PMC1934899

[B200] Quast R. B. Mrusek D. Hoffmeister C. Sonnabend A. Kubick S. (2015). Cotranslational incorporation of non-standard amino acids using cell-free protein synthesis. FEBS Lett. 589, 1703–1712. 10.1016/j.febslet.2015.04.041 25937125

[B201] Quast R. B. Sonnabend A. Stech M. Wüstenhagen D. A. Kubick S. (2016). High-yield cell-free synthesis of human EGFR by IRES-mediated protein translation in a continuous exchange cell-free reaction format. Sci. Rep. 6, 30399. 10.1038/srep30399 27456041 PMC4960648

[B202] Quinn C. M. Wang M. Fritz M. P. Runge B. Ahn J. Xu C. (2018). Dynamic regulation of HIV-1 capsid interaction with the restriction factor TRIM5α identified by magic-angle spinning NMR and molecular dynamics simulations. Proc. Natl. Acad. Sci. U.S.A. 115, 11519–11524. 10.1073/pnas.1800796115 30333189 PMC6233135

[B203] Ramadan A. Nemoto K. Seki M. Shinozaki K. Takeda H. Takahashi H. (2015). Wheat germ-based protein libraries for the functional characterisation of the Arabidopsis E2 ubiquitin conjugating enzymes and the RING-type E3 ubiquitin ligase enzymes. BMC Plant Biol. 15, 275. 10.1186/s12870-015-0660-9 26556605 PMC4641371

[B204] Ranaghan M. J. Schwall C. T. Alder N. N. Birge R. R. (2011). Green proteorhodopsin reconstituted into nanoscale phospholipid bilayers (nanodiscs) as photoactive monomers. J. Am. Chem. Soc. 133, 18318–18327. 10.1021/ja2070957 21951206 PMC3218432

[B205] Rancy P. C. Thorpe C. (2008). Oxidative protein folding *in vitro*: a study of the cooperation between quiescin-sulfhydryl oxidase and protein disulfide isomerase. Biochemistry 47, 12047–12056. 10.1021/bi801604x 18937500 PMC2892342

[B206] Rawlings A. E. (2018). Membrane protein engineering to the rescue. Biochem. Soc. Trans. 46, 1541–1549. 10.1042/BST20180140 30381335

[B207] Reece-Hoyes J. S. Walhout A. J. M. (2018). Gateway recombinational cloning. Cold Spring Harb Protoc. 12, 114. 10.1101/pdb.top094912 PMC5935001 29295908

[B208] Revathi B. R. K. V. A. Hinnebusch A. G. Bhuyan A. K. Ksr S. Sudhakar A. (2010). Trends in wheat germ cell free protein expression system with an emphasis on up-scaling and industrial application. Indian J. Sci. Technol. 3, 349–354.

[B209] Rigaud J. L. (2002). Membrane proteins: functional and structural studies using reconstituted proteoliposomes and 2-D crystals. Braz. J. Med. Biol. Res. 35, 753–766. 10.1590/s0100-879x2002000700001 12131914

[B210] Ritchie T. K. Grinkova Y. V. Bayburt T. H. Denisov I. G. Zolnerciks J. K. Atkins W. M. (2009). Chapter 11 - reconstitution of membrane proteins in phospholipid bilayer nanodiscs. Meth Enzymol. 464, 211–231. 10.1016/S0076-6879(09)64011-8 PMC4196316 19903557

[B211] Roberts B. E. Paterson B. M. (1973). Efficient translation of tobacco mosaic virus RNA and rabbit globin 9S RNA in a cell-free system from commercial wheat germ. Proc. Natl. Acad. Sci. U.S.A. 70, 2330–2334. 10.1073/pnas.70.8.2330 4525168 PMC433729

[B212] Romanov V. Davidoff S. N. Miles A. R. Grainger D. W. Gale B. K. Brooks B. D. (2014). A critical comparison of protein microarray fabrication technologies. Analyst 139, 1303–1326. 10.1039/c3an01577g 24479125

[B213] Rosenberg R. L. East J. E. (1992). Cell-free expression of functional Shaker potassium channels. Nature 360, 166–169. 10.1038/360166a0 1436093

[B214] Rosenblum G. Cooperman B. S. (2014). Engine out of the chassis: cell-free protein synthesis and its uses. FEBS Lett. 588, 261–268. 10.1016/j.febslet.2013.10.016 24161673 PMC4133780

[B215] Saaranen M. J. Ruddock L. W. (2019). Applications of catalyzed cytoplasmic disulfide bond formation. Biochem. Soc. Trans. 47, 1223–1231. 10.1042/BST20190088 31671179

[B216] Sachse R. Dondapati S. K. Fenz S. F. Schmidt T. Kubick S. (2014). Membrane protein synthesis in cell-free systems: from bio-mimetic systems to bio-membranes, FEBS Lett. 588, 2774–2781. 10.1016/j.febslet.2014.06.007 24931371

[B217] Saint-Léger A. Bello C. Dans P. D. Torres A. G. Novoa E. M. Camacho N. (2016). Saturation of recognition elements blocks evolution of new tRNA identities. Sci. Adv. 2, e1501860. 10.1126/sciadv.1501860 27386510 PMC4928997

[B218] Salehi A. S. Earl C. C. Muhlestein C. Bundy B. C. (2016). Escherichia coli-based cell-free extract development for protein-based cancer therapeutic production. Int. J. Dev. Biol. 60, 237–243. 10.1387/ijdb.160125bb 27251070

[B219] Samuel P. P. Smith L. P. Phillips G. N. Jr. Olson J. S. (2015). Apoglobin stability is the major factor governing both cell-free and *in Vivo* expression of holomyoglobin. J. Biol. Chem. 290, 23479–23495. 10.1074/jbc.M115.672204 26205820 PMC4583012

[B220] Sano N. Rajjou L. North H. M. (2020). Lost in translation: physiological roles of stored mRNAs in seed germination. Plants 9, 119. 10.3390/plants9030347 PMC7154877 32164149

[B221] Sansuk K. Balog C. I. Van Der Does A. M. Booth R. De Grip W. J. Deelder A. M. (2008). GPCR proteomics: mass spectrometric and functional analysis of histamine H1 receptor after baculovirus-driven and *in vitro* cell free expression. J. Proteome Res. 7, 621–629. 10.1021/pr7005654 18177001

[B222] Sawasaki T. Hasegawa Y. Tsuchimochi M. Kamura N. Ogasawara T. Kuroita T. (2002a). A bilayer cell-free protein synthesis system for high-throughput screening of gene products. FEBS Lett. 514, 102–105. 10.1016/s0014-5793(02)02329-3 11904190

[B223] Sawasaki T. Ogasawara T. Morishita R. Endo Y. (2002b). A cell-free protein synthesis system for high-throughput proteomics. Proc. Natl. Acad. Sci. U.S.A. 99, 14652–14657. 10.1073/pnas.232580399 12409616 PMC137474

[B224] Schinn S. M. Broadbent A. Bradley W. T. Bundy B. C. (2016). Protein synthesis directly from PCR: progress and applications of cell-free protein synthesis with linear DNA. N. Biotechnol. 33, 480–487. 10.1016/j.nbt.2016.04.002 27085957

[B225] Schmidt T. G. Batz L. Bonet L. Carl U. Holzapfel G. Kiem K. (2013). Development of the Twin-Strep-tag® and its application for purification of recombinant proteins from cell culture supernatants. Protein Expr. Purif. 92, 54–61. 10.1016/j.pep.2013.08.021 24012791

[B226] Schmidt T. G. Skerra A. (2007). The Strep-tag system for one-step purification and high-affinity detection or capturing of proteins. Nat. Protoc. 2, 1528–1535. 10.1038/nprot.2007.209 17571060

[B227] Schneider B. Junge F. Shirokov V. A. Durst F. Schwarz D. Dötsch V. (2009). Membrane protein expression in cell-free systems. Totowa, NJ: Humana Press, 165–186. 10.1007/978-1-60761-344-2_11 20099146

[B228] Schwarz D. Dötsch V. Bernhard F. (2008). Production of membrane proteins using cell-free expression systems. Proteomics 8, 3933–3946. 10.1002/pmic.200800171 18763710

[B229] Seddon A. M. Curnow P. Booth P. J. (2004). Membrane proteins, lipids and detergents: not just a soap opera. Biochim. Biophys. Acta 1666, 105–117. 10.1016/j.bbamem.2004.04.011 15519311

[B230] Seki E. Matsuda N. Kigawa T. (2009). Multiple inhibitory factor removal from an Escherichia coli cell extract improves cell-free protein synthesis. J. Biosci. Bioeng. 108, 30–35. 10.1016/j.jbiosc.2009.02.011 19577188

[B231] Sevova E. S. Goren M. A. Schwartz K. J. Hsu F. F. Turk J. Fox B. G. (2010). Cell-free synthesis and functional characterization of sphingolipid synthases from parasitic trypanosomatid protozoa. J. Biol. Chem. 285, 20580–20587. 10.1074/jbc.M110.127662 20457606 PMC2898309

[B232] Shadiac N. Nagarajan Y. Waters S. Hrmova M. (2013). Close allies in membrane protein research: cell-free synthesis and nanotechnology. Mol. Membr. Biol. 30, 229–245. 10.3109/09687688.2012.762125 23343215

[B233] Shergalis A. G. Hu S. Bankhead A. 3rd Neamati N. (2020). Role of the ERO1-PDI interaction in oxidative protein folding and disease. Pharmacol. Ther. 210, 107525. 10.1016/j.pharmthera.2020.107525 32201313 PMC7316501

[B234] Silverman A. D. Karim A. S. Jewett M. C. (2020). Cell-free gene expression: an expanded repertoire of applications. Nat. Rev. Genet. 21, 151–170. 10.1038/s41576-019-0186-3 31780816

[B235] Singh S. Springer M. Steen J. Kirschner M. W. Steen H. (2009). FLEXIQuant: a novel tool for the absolute quantification of proteins, and the simultaneous identification and quantification of potentially modified peptides. J. Proteome Res. 8, 2201–2210. 10.1021/pr800654s 19344176 PMC2868505

[B236] Smith M. T. Berkheimer S. D. Werner C. J. Bundy B. C. (2014). Lyophilized Escherichia coli-based cell-free systems for robust, high-density, long-term storage. BioTechniques 56, 186–193. 10.2144/000114158 24724844

[B237] Spice A. J. Aw R. Bracewell D. G. Polizzi K. M. (2020). Improving the reaction mix of a Pichia pastoris cell-free system using a design of experiments approach to minimise experimental effort. Synth. Syst. Biotechnol. 5, 137–144. 10.1016/j.synbio.2020.06.003 32637667 PMC7320237

[B238] Spirin A. S. Baranov V. I. Ryabova L. A. Ovodov S. Y. Alakhov Y. B. (1988). A continuous cell-free translation system capable of producing polypeptides in high yield. Science 242, 1162–1164. 10.1126/science.3055301 3055301

[B239] Spirin A. S. (2004). High-throughput cell-free systems for synthesis of functionally active proteins. Trends Biotechnol. 22, 538–545. 10.1016/j.tibtech.2004.08.012 15450748

[B240] Stark J. C. Huang A. Hsu K. J. Dubner R. S. Forbrook J. Marshalla S. (2019). BioBits health: classroom Activities exploring engineering, biology, and human health with fluorescent readouts. ACS Synth. Biol. 8, 1001–1009. 10.1021/acssynbio.8b00381 30925042

[B241] Stech M. Hust M. Schulze C. Dübel S. Kubick S. (2014). Cell-free eukaryotic systems for the production, engineering, and modification of scFv antibody fragments. Eng. Life Sci. 14, 387–398. 10.1002/elsc.201400036 25821419 PMC4374706

[B242] Stueber D. Ibrahimi I. Cutler D. Dobberstein B. Bujard H. (1984). A novel *in vitro* transcription-translation system: accurate and efficient synthesis of single proteins from cloned DNA sequences. EMBO J. 3, 3143–3148. 10.1002/j.1460-2075.1984.tb02271.x 6526014 PMC557830

[B243] Sullivan C. J. Pendleton E. D. Sasmor H. H. Hicks W. L. Farnum J. B. Muto M. (2016). A cell-free expression and purification process for rapid production of protein biologics. Biotechnol. J. 11, 238–248. 10.1002/biot.201500214 26427345

[B244] Sun M. A. Wang Y. Zhang Q. Xia Y. Ge W. Guo D. (2017). Prediction of reversible disulfide based on features from local structural signatures. BMC Genomics 18, 279. 10.1186/s12864-017-3668-8 28376774 PMC5379614

[B245] Suzuki K. Inoue H. Matsuoka S. Tero R. Hirano-Iwata A. Tozawa Y. (2020). Establishment of a cell-free translation system from rice callus extracts. Biosci. Biotechnol. Biochem. 112, 1–9. 10.1080/09168451.2020.1779024 32543982

[B246] Suzuki Y. (2018). Functional G-protein-coupled receptor (GPCR) synthesis: the Pharmacological analysis of human h vistamine H1 receptor (HRH1) synthesized by a wheat germ cell-free protein synthesis system com vbined with Asolectin Glycerosomes, Berlin: Springer, 1–13. 10.3389/fphar.2018.00038 PMC5808195 29467651

[B247] Takahashi H. Takahashi C. Moreland N. J. Chang Y. T. Sawasaki T. Ryo A. (2012). Establishment of a robust dengue virus NS3-NS5 binding assay for identification of protein-protein interaction inhibitors. Antivir. Res 96, 305–314. 10.1016/j.antiviral.2012.09.023 23072882

[B248] Takahashi H. Uematsu A. Yamanaka S. Imamura M. Nakajima T. Doi K. (2016). Establishment of a wheat cell-free synthesized protein array containing 250 human and mouse E3 ubiquitin ligases to identify novel interaction between E3 ligases and substrate proteins. PLoS ONE 11, e0156718. 10.1371/journal.pone.0156718 27249653 PMC4889105

[B249] Takahashi H. Yamanaka S. Kuwada S. Higaki K. Kido K. Sato Y. (2020). A human DUB protein array for clarification of linkage specificity of polyubiquitin chain and application to evaluation of its inhibitors. Biomedicines 8, 114. 10.3390/biomedicines8060152 PMC7344921 32512835

[B250] Takai K. Sawasaki T. Endo Y. (2010). Practical cell-free protein synthesis system using purified wheat embryos. Nat. Protoc. 5, 227–238. 10.1038/nprot.2009.207 20134421

[B251] Takeda H. Zhou W. Kido K. Suno R. Iwasaki T. Kobayashi T. (2017). CP5 system, for simple and highly efficient protein purification with a C-terminal designed mini tag. PLoS ONE 12, e0178246. 10.1371/journal.pone.0178246 28542437 PMC5444806

[B252] Takemori N. Takemori A. Tanaka Y. Endo Y. Hurst J. L. Gómez-Baena G. (2017). MEERCAT: multiplexed efficient cell free expression of recombinant QconCATs for large scale Absolute proteome quantification. Mol. Cel Proteomics 16, 2169–2183. 10.1074/mcp.RA117.000284 PMC5724179 29055021

[B253] Takemori N. Takemori A. Tanaka Y. Ishizaki J. Hasegawa H. Shiraishi A. (2016). High-throughput production of a stable isotope-labeled peptide library for targeted proteomics using a wheat germ cell-free synthesis system. Mol. Biosyst. 12, 2389–2393. 10.1039/c6mb00209a 27203355

[B254] Thavarajah W. Silverman A. D. Verosloff M. S. Kelley-Loughnane N. Jewett M. C. Lucks J. B. (2020). Point-of-Use detection of environmental fluoride via a cell-free riboswitch-based biosensor. ACS Synth. Biol. 9, 10–18. 10.1021/acssynbio.9b00347 31829623 PMC7412506

[B255] Thoring L. Wüstenhagen D. A. Borowiak M. Stech M. Sonnabend A. Kubick S. (2016). Cell-free systems based on CHO cell lysates: optimization strategies, synthesis of “Difficult-to-Express” proteins and future perspectives. PLoS ONE 11, e0163670. 10.1371/journal.pone.0163670 27684475 PMC5042383

[B256] Timm A. C. Shankles P. G. Foster C. M. Doktycz M. J. Retterer S. T. (2016). Toward microfluidic reactors for cell-free protein synthesis at the point-of-care. Small 12, 810–817. 10.1002/smll.201502764 26690885

[B257] Tinafar A. Jaenes K. Pardee K. (2019). Synthetic biology goes cell-free. BMC Biol. 17, 64. 10.1186/s12915-019-0685-x 31395057 PMC6688370

[B258] Tonelli M. Singarapu K. K. Makino S. Sahu S. C. Matsubara Y. Endo Y. (2011). Hydrogen exchange during cell-free incorporation of deuterated amino acids and an approach to its inhibition. J. Biomol. NMR 51, 467–476. 10.1007/s10858-011-9575-4 21984356 PMC3254145

[B259] Tugarinov V. Kanelis V. Kay L. E. (2006). Isotope labeling strategies for the study of high-molecular-weight proteins by solution NMR spectroscopy. Nat. Protoc. 1, 749–754. 10.1038/nprot.2006.101 17406304

[B260] Tyler R. C. Aceti D. J. Bingman C. A. Cornilescu C. C. Fox B. G. Frederick R. O. (2005). Comparison of cell-based and cell-free protocols for producing target proteins from the *Arabidopsis thaliana* genome for structural studies. Proteins 59, 633–643. 10.1002/prot.20436 15789406

[B261] Venkat S. Chen H. Gan Q. Fan C. (2019). The application of cell-free protein synthesis in genetic code expansion for post-translational modifications. Front. Pharmacol. 10, 248. 10.3389/fphar.2019.00248 30949051 PMC6436179

[B262] Verma M. Choi J. Cottrell K. A. Lavagnino Z. Thomas E. N. Pavlovic-Djuranovic S. (2019). A short translational ramp determines the efficiency of protein synthesis. Nat. Commun. 10, 5774. 10.1038/s41467-019-13810-1 31852903 PMC6920384

[B263] Vinarov D. A. Loushin Newman C. L. Markley J. L. (2006a). Wheat germ cell-free platform for eukaryotic protein production. FEBS J. 273, 4160–4169. 10.1111/j.1742-4658.2006.05434.x 16930128

[B264] Vinarov D. A. Lytle B. L. Peterson F. C. Tyler E. M. Volkman B. F. Markley J. L. (2004). Cell-free protein production and labeling protocol for NMR-based structural proteomics. Nat. Methods 1, 149–153. 10.1038/nmeth716 15782178

[B265] Vinarov D. A. Markley J. L. (2014). High-throughput automated platform for nuclear magnetic resonance-based structural proteomics. Expert Rev. Proteomics 2, 49–55. 10.1586/14789450.2.1.49 15966852

[B266] Vinarov D. A. Newman C. L. Tyler E. M. Markley J. L. Shahan M. N. (2006b). Wheat germ cell-free expression system for protein production current protocols in protein science/editorial board, Curr. Protoc. Protein Sci. 11, 121. 10.1002/0471140864.ps0518s44 18429309

[B267] Wang L. Tonggu L. (2015). Membrane protein reconstitution for functional and structural studies. Sci. China Life Sci. 58, 66–74. 10.1007/s11427-014-4769-0 25576454

[B268] Wang M. Quinn C. M. Perilla J. R. Zhang H. Shirra R. Jr. Hou G. (2017). Quenching protein dynamics interferes with HIV capsid maturation. Nat. Commun. 8, 1779. 10.1038/s41467-017-01856-y 29176596 PMC5701193

[B269] Wang S. Fogeron M. L. Schledorn M. Dujardin M. Penzel S. Burdette D. (2019). Combining cell-free protein synthesis and NMR into a tool to study capsid assembly modulation. Front. Mol. Biosci. 6, 67. 10.3389/fmolb.2019.00067 31440516 PMC6694763

[B270] Watanabe M. Miyazono K. Tanokura M. Sawasaki T. Endo Y. Kobayashi I. (2010). Cell-free protein synthesis for structure determination by X-ray crystallography. Methods Mol. Biol. 607, 149–160. 10.1007/978-1-60327-331-2_13 20204855

[B271] Waugh D. S. (2011). An overview of enzymatic reagents for the removal of affinity tags. Protein Expr. Purif. 80, 283–293. 10.1016/j.pep.2011.08.005 21871965 PMC3195948

[B272] Wiedemann C. Kumar A. Lang A. Ohlenschläger O. (2020). Cysteines and disulfide bonds as structure-forming units: insights from different domains of life and the potential for characterization by NMR. Front. Chem. 8, 280. 10.3389/fchem.2020.00280 32391319 PMC7191308

[B273] Wingfield P. T. (2015). Overview of the purification of recombinant proteins. Curr. Protoc. Protein Sci. 80 **,** 6–35. 10.1002/0471140864.ps0601s80 25829302 PMC4410719

[B274] Winther J. R. Thorpe C. (2014). Quantification of thiols and disulfides. Biochim. Biophys. Acta 1840, 838–846. 10.1016/j.bbagen.2013.03.031 23567800 PMC3766385

[B275] Witte K. Kaiser A. Schmidt P. Splith V. Thomas L. Berndt S. (2013). Oxidative *in vitro* folding of a cysteine deficient variant of the G protein-coupled neuropeptide Y receptor type 2 improves stability at high concentration. Biol. Chem. 394, 1045–1056. 10.1515/hsz-2013-0120 23732681

[B276] Wood D. W. (2014). New trends and affinity tag designs for recombinant protein purification. Curr. Opin. Struct. Biol. 26, 54–61. 10.1016/j.sbi.2014.04.006 24859434

[B277] Wu P. S. Ozawa K. Lim S. P. Vasudevan S. G. Dixon N. E. Otting G. (2007). Cell-free transcription/translation from PCR-amplified DNA for high-throughput NMR studies. Angew. Chem. Int. Ed. Engl. 46, 3356–3358. 10.1002/anie.200605237 17378006

[B278] Wu Y. Wang Z. Qiao X. Li J. Shu X. Qi H. (2020). Emerging methods for efficient and extensive incorporation of non-canonical amino acids using cell-free systems. Front. Bioeng. Biotechnol. 8, 863. 10.3389/fbioe.2020.00863 32793583 PMC7387428

[B279] Yadav D. K. Yadav N. Yadav S. Haque S. Tuteja N. (2016). An insight into fusion technology aiding efficient recombinant protein production for functional proteomics. Arch. Biochem. Biophys. 612, 57–77. 10.1016/j.abb.2016.10.012 27771300

[B280] Yamaoka Y. Matsuyama S. Fukushi S. Matsunaga S. Matsushima Y. Kuroyama H. (2016). Development of monoclonal antibody and diagnostic test for Middle East respiratory syndrome coronavirus using cell-free synthesized nucleocapsid antigen. Front. Microbiol. 7, 509. 10.3389/fmicb.2016.00509 27148198 PMC4837155

[B281] Yamaoka Y. Jeremiah S. S. Miyakawa K. Saji R. Nishii M. Takeuchi I. (2020). Whole nucleocapsid protein of SARS-CoV-2 may cause false positive results in serological assays. Clin. Infect. Dis. 11, 33. 10.1093/cid/ciaa637 PMC7314131 32445559

[B282] Yano T. Takeda H. Uematsu A. Yamanaka S. Nomura S. Nemoto K. (2016). AGIA tag system based on a high affinity rabbit monoclonal antibody against human dopamine receptor D1 for protein analysis. PLoS ONE 11, e0156716. 10.1371/journal.pone.0156716 27271343 PMC4894603

[B283] Yin G. Swartz J. R. (2004). Enhancing multiple disulfide bonded protein folding in a cell-free system. Biotechnol. Bioeng. 86, 188–195. 10.1002/bit.10827 15052638

[B284] Yu C. H. Dang Y. Zhou Z. Wu C. Zhao F. Sachs M. S. (2015). Codon usage influences the local rate of translation elongation to regulate Co-translational protein folding. Mol. Cel. 59, 744–754. 10.1016/j.molcel.2015.07.018 PMC4561030 26321254

[B285] Zárate X. Galbraith D. W. (2014). A cell-free expression platform for production of protein microarrays. Methods Mol. Biol. 1118, 297–307. 10.1007/978-1-62703-782-2_21 24395426

[B286] Zawada J. F. Yin G. Steiner A. R. Yang J. Naresh A. Roy S. M. (2011). Microscale to manufacturing scale-up of cell-free cytokine production—a new approach for shortening protein production development timelines. Biotechnol. Bioeng. 108, 1570–1578. 10.1002/bit.23103 21337337 PMC3128707

[B287] Zemella A. Thoring L. Hoffmeister C. Kubick S. (2015). Cell-free protein synthesis: pros and cons of prokaryotic and eukaryotic systems. Chembiochem 16, 2420–2431. 10.1002/cbic.201500340 26478227 PMC4676933

[B288] Zhang H. Hou G. Lu M. Ahn J. Byeon I. L. Langmead C. J. (2016). HIV-1 capsid function is regulated by dynamics: quantitative atomic-resolution insights by integrating magic-angle-Angle-Spinning NMR, QM/MM, and MD. J. Am. Chem. Soc. 138, 14066–14075. 10.1021/jacs.6b08744.s001 27701859 PMC5380593

[B289] Zhang Y. Nunoura T. Nishiura D. Hirai M. Shimamura S. Kurosawa K. (2020). A single-molecule counting approach for convenient and ultrasensitive measurement of restriction digest efficiencies. PLoS ONE 15, e0244464. 10.1371/journal.pone.0244464 33382779 PMC7775078

[B290] Zhao L. Nishiura D. Hirai M. Shimamura S., (2010). Engineering of a wheat germ expression system to provide compatibility with a high throughput pET-based cloning platform. J Struct Funct Genomics 11, 201–209. 10.1007/s10969-010-9093-8 20574660 PMC2921493

[B291] Zhou W. Takeda H. (2020). Cell-free production of proteoliposomes for functional analysis and antibody development targeting membrane proteins. J. Vis. Exp. 21, 127–133. 10.3791/61871 33044457

[B292] Zhu B. Mizoguchi T. Kojima T. Nakano H. (2015). Ultra-high-throughput screening of an in vitro-synthesized horseradish peroxidase displayed on microbeads using cell sorter. PLoS ONE 10, e0127479. 10.1371/journal.pone.0127479 25993095 PMC4439038

[B293] Zhu B. Gan R. Cabezas M. D. Kojima T. Nicol R. Jewett M. C. (2020). Increasing cell-free gene expression yields from linear templates in Escherichia coli and Vibrio natriegens extracts by using DNA-binding proteins. Biotechnol. Bioeng. 12, 157. 10.1101/2020.07.22.214221 32816360

[B294] Zhu G. Zhu C. Zhu Y. Sun F. (2020). Minireview of progress in the structural study of SARS-CoV-2 proteins. Curr. Res. Microb. Sci. 1, 53–61. 10.1016/j.crmicr.2020.06.003 33236001 PMC7323663

[B295] Zucchelli S. Cotella D. Takahashi H. Carrieri C. Cimatti L. Fasolo F. (2015a). SINEUPs: a new class of natural and synthetic antisense long non-coding RNAs that activate translation. RNA Biol. 12, 771–779. 10.1080/15476286.2015.1060395 26259533 PMC4615742

[B296] Zucchelli S. Fasolo F. Russo R. Cimatti L. Patrucco L. Takahashi H. (2015b). SINEUPs are modular antisense long non-coding RNAs that increase synthesis of target proteins in cells. Front Cel Neurosci. 9, 174. 10.3389/fncel.2015.00174 PMC4429562 26029048

[B297] Zuker M. (2003). Mfold web server for nucleic acid folding and hybridization prediction. Nucleic Acids Res. 31, 3406–3415. 10.1093/nar/gkg595 12824337 PMC169194

